# Intranasal Immunization with Acellular Pertussis Vaccines Results in Long-Term Immunity to Bordetella pertussis in Mice

**DOI:** 10.1128/IAI.00607-20

**Published:** 2021-02-16

**Authors:** M. Allison Wolf, Dylan T. Boehm, Megan A. DeJong, Ting Y. Wong, Emel Sen-Kilic, Jesse M. Hall, Catherine B. Blackwood, Kelly L. Weaver, Claire O. Kelly, Caleb A. Kisamore, Graham J. Bitzer, Justin R. Bevere, Mariette Barbier, F. Heath Damron

**Affiliations:** a Department of Microbiology, Immunology, and Cell Biology, West Virginia University, Morgantown, West Virginia, USA; b Vaccine Development Center at West Virginia University Health Sciences Center, Morgantown, West Virginia, USA; Washington State University

**Keywords:** *Bordetella pertussis*, adjuvants, beta-glucan particle, intranasal pertussis vaccine, mucosal vaccines, pertussis toxin, whooping cough

## Abstract

Bordetella pertussis colonizes the respiratory mucosa of humans, inducing an immune response seeded in the respiratory tract. An individual, once convalescent, exhibits long-term immunity to the pathogen. Current acellular pertussis (aP) vaccines do not induce the long-term immune response observed after natural infection in humans. In this study, we evaluated the durability of protection from intranasal (i.n.) pertussis vaccines in mice. Mice that convalesced from B. pertussis infection served as a control group. Mice were immunized with a mock vaccine (phosphate-buffered saline [PBS]), aP only, or an aP base vaccine combined with one of the following adjuvants: alum, curdlan, or purified whole glucan particles (IRI-1501). We utilized two study designs: short term (challenged 35 days after priming vaccination) and long term (challenged 6 months after boost). The short-term study demonstrated that immunization with i.n. vaccine candidates decreased the bacterial burden in the respiratory tract, reduced markers of inflammation, and induced significant serum and lung antibody titers. In the long-term study, protection from bacterial challenge mirrored the results observed in the short-term challenge study. Immunization with pertussis antigens alone was surprisingly protective in both models; however, the alum and IRI-1501 adjuvants induced significant B. pertussis-specific IgG antibodies in both the serum and lung and increased numbers of anti-B. pertussis IgG-secreting plasma cells in the bone marrow. Our data indicate that humoral responses induced by the i.n. vaccines correlated with protection, suggesting that long-term antibody responses can be protective.

## Abstract (precis)

Bordetella pertussis colonizes the respiratory mucosa of humans, inducing an immune response seeded in the respiratory tract. An individual, once convalescent, exhibits long-term immunity to the pathogen.

## INTRODUCTION

Pertussis is a highly contagious disease caused by infection of the upper respiratory tract by the pathogen Bordetella pertussis. Prior to the first pertussis vaccine, the United States averaged around 200,000 cases per year and thousands of infant deaths (1). A whole-cell pertussis vaccine that included the tetanus and diphtheria toxoids (DTP) was introduced in the 1940s, and the numbers of annual cases and deaths decreased profoundly (2). After reactogenicity concerns, DTP was replaced with an acellular formulation (DTaP) in the United States and Europe, and since then, the average number of yearly cases has increased (1, 3, 4). In the United States, epidemics were observed in 2004 (25,827 cases), 2010 (27,550 cases), and 2012 (48,277 cases) (1). The worldwide incidence is still high, with approximately 24.1 million cases in 2014, which includes 160,700 deaths in children 5 years of age and younger (3). Studies report an increase in the pertussis incidence among fully vaccinated children and adults, indicating that there is incomplete protection for vaccinated individuals (1, 5, 6). It is also hypothesized that asymptomatic carriage among DTaP-vaccinated individuals may contribute to the increased number of cases and leave those unvaccinated at risk (4). A study performed in infant baboons observed asymptomatic carriage after B. pertussis challenge in a group that was immunized with DTaP, and while clinical symptoms of pertussis were not observed, colonization and transmission were similar to those in naive baboons (7). Conversely, convalescent baboons were not colonized after rechallenge, suggesting more complete protection (7). Furthermore, it has been reported that convalescence in humans can confer long-term protection for 20 years, whereas DTaP immunity averages 3 years, further supporting the lasting protection afforded by natural infection (8).

It has been postulated that the longevity of protection in convalescent individuals is associated with mucosal immunity (9–11). Pertussis occurs when B. pertussis attaches to the mucosal cells in the respiratory tract, which in turn induces a mucosal immune response that primes the respiratory tract to protect against subsequent infections (9). Recently, mucosal immunization has been of increased interest. Previous studies demonstrated the induction of strong mucosal immune responses after intranasal (i.n.) immunization with a formalin-inactivated whole-cell pertussis vaccine (WCV) in adults and oral vaccination using heat-inactivated WCV in infants (12, 13). A recent preclinical mucosal vaccination study using a novel adjuvant LP-GMP (a combination of an intracellular receptor stimulator of interferon gene [STING] agonist and a ligand of Toll-like receptor 2 [TLR2]) combined with an acellular vaccine as well as an additional study using outer membrane vesicles (OMVs) of pertussis vaccine (omvPV) demonstrated that i.n. immunization with a pertussis vaccine can confer protection from B. pertussis challenge (14, 15). A live attenuated vaccine, BPZE1, has also exhibited protection in preclinical models and has progressed to clinical trials (16–19).

Previously, our laboratory also showed that i.n. vaccination can elicit a protective immune response in a murine challenge model (20). We added a novel adjuvant, curdlan, to DTaP in order to study the mucosal immune response after i.n. vaccination. Curdlan, a 1,3-β-glucan, was selected because it can prompt a Th1/Th17 response (21). Th1/Th17 polarization occurs both after DTP vaccination and with natural infection, and this induction is correlated with prolonged protection in several animal models (22–26). i.n. immunization with DTaP, with or without curdlan, decreased the respiratory bacterial burden, but i.n. DTaP with curdlan increased interleukin-17a (IL-17a) in the lung compared to i.n. DTaP alone and the combination of curdlan with DTaP also increased IgA levels in the respiratory tract (20). Additionally, DTaP with curdlan was retained in the nasopharyngeal cavity, as demonstrated by imaging and cytometric analyses (20). Overall, that study demonstrated that i.n. DTaP formulations provided protection against B. pertussis challenge and that novel adjuvants may alter the mucosal immune response.

The current study aimed at further evaluating the effects of adjuvants on an acellular i.n. pertussis vaccine. Alum has long been considered the standard with regard to vaccine adjuvants and is found in the current DTaP vaccine, but novel adjuvants may increase immunity and prolong protection (27, 28). For the present study, we deconstructed and formulated an experimental acellular base vaccine (aP) that mimics the 1/20 antigen mass found in the current human DTaP vaccine, which contains 25 μg of pertussis toxin (PT), 25 μg of filamentous hemagglutinin (FHA), and 8 μg of pertactin (PRN), to better elucidate the role that individual adjuvants have in i.n. aP immunity. The study was designed to evaluate the currently used adjuvant, alum, as well as two β-glucans (curdlan and IRI-1501). We selected β-glucans for this study based on the highly promising protection data obtained in our previous study, which suggested that β-glucans such as curdlan can elicit a Th17 response against pertussis and help retain the vaccine at the nares (20). β-Glucans are derived from fungi and yeast and are reported to increase the immune response due in part to their structural similarity to natural pathogens (10, 21, 29, 30). IRI-1501 is a novel 2- to 4-μm whole β-glucan particle (WGP) derived from Saccharomyces cerevisiae and can induce a Dectin-1-mediated response in murine models (31). The activation of this receptor can increase the uptake of antigen by phagocytic cells and induce proinflammatory cytokine release, leading to T-cell polarization (21, 28, 31). β-Glucans like curdlan and IRI-1501 are exciting adjuvants to explore for a mucosal pertussis vaccine due to their ability to increase antigen uptake by M cells as well as their proposed ability to train human immunity through functional reprogramming of innate immune cells (32). Most pertussis researchers would agree that a vaccine candidate capable of stimulating a mucosal immune response could induce longer-lasting immunity similar to what is seen with both natural infection and convalescence.

In the current study, the i.n. aP vaccines provide protection from B. pertussis colonization that is equal to the protection observed in the convalescent group. Together, our data suggest that the β-glucan IRI-1501 elicited a superior humoral immune response, but convalescent mice induced more of a Th1/Th17 immune response. Collectively, these data indicate that in a murine model, acellular i.n. vaccine formulations can stimulate a mucosal immune response and offer long-lasting protection from B. pertussis challenge.

## RESULTS

### Intranasal immunization reduced the B. pertussis bacterial burden in the respiratory tract after a short-term immunization study.

We used a murine vaccination-and-challenge model to compare adjuvanted (alum, curdlan, or IRI-1501) aP vaccines to a nonadjuvanted aP vaccine as well as to compare these experimental vaccines to mock-vaccinated and convalescent controls. We hypothesized that combining adjuvants with our experimental acellular vaccine would protect BALB/c mice against B. pertussis challenge. Our acellular base vaccine contained 1/20 of the reported PT, FHA, and PRN concentrations in the DTaP vaccine. Current DTaP vaccines contain chemically detoxified PT (dPT). However, in our base vaccine, we elected to use genetically detoxified PT (gPT) as clinical evidence suggests that gPT is a superior immunogen (33, 34). gPT is produced in strains that have point mutations in the *ptxA* gene that result in 2 amino acid substitutions that abrogate enzymatic activity. Without the need to cross-link by formaldehyde, B-cell epitopes are not destroyed, and robust PT-neutralizing antibodies are generated compared to dPT (33, 35).

**FIG 1:**
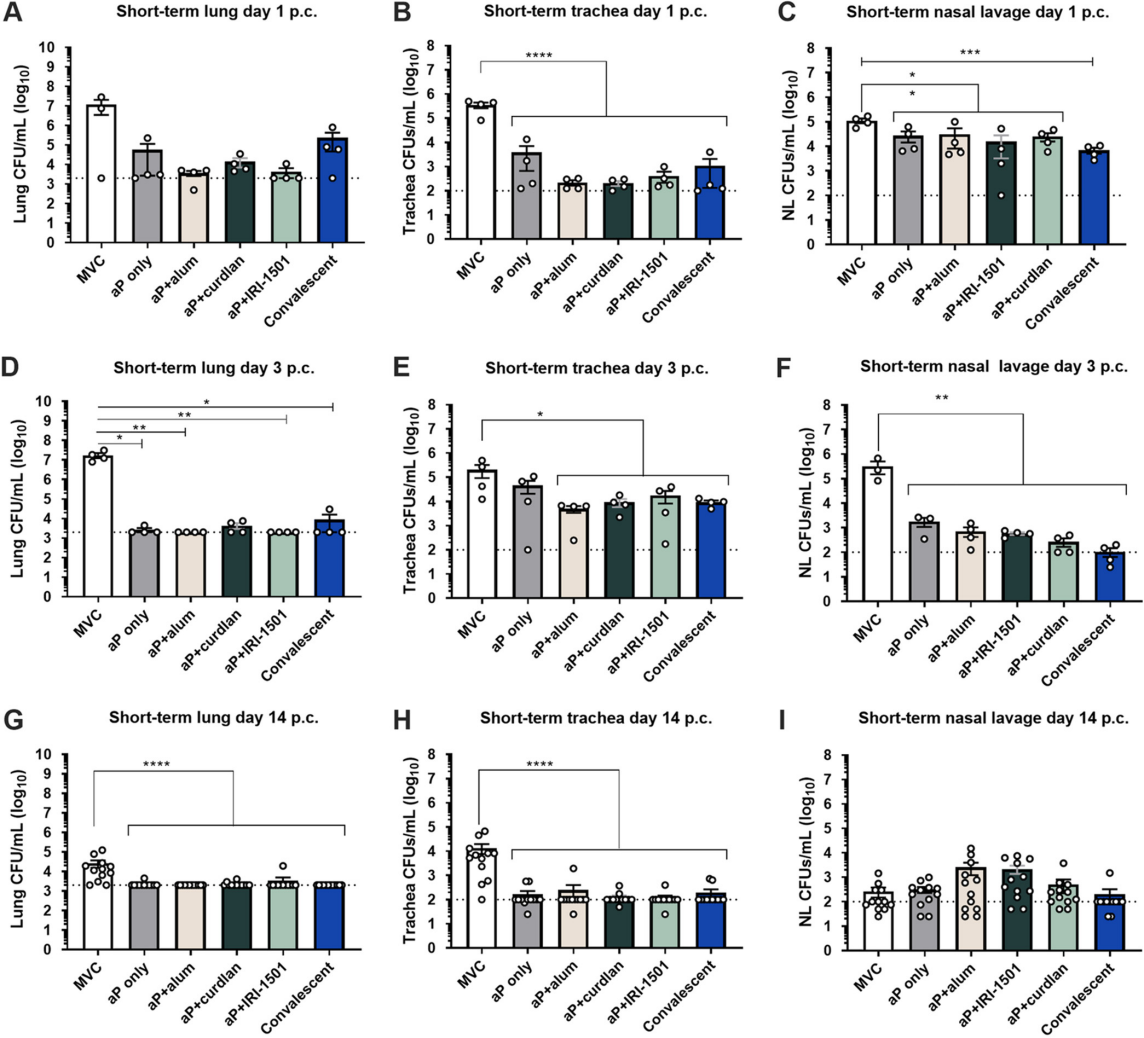
In the short-term study, the bacterial burden in the respiratory tract (lung, trachea, and nasal lavage fluid [NL]) was enumerated 1, 3, and 14 days after B. pertussis challenge as determined by CFU. B. pertussis was quantified by counting serially diluted CFU following challenge. CFU counts were determined from lung homogenates (A, D, and G), trachea homogenates (B, E, and H), and nasal lavage fluid (C, F, and I). Data are presented as means ± standard errors of the means (SEM) (*n* = 4 per treatment group for days 1 and 3 postchallenge [p.c.] and *n* = 14 per treatment group for day 14 postchallenge, with four averaged technical replicates). The dotted line indicates the lowest limit of detection. One-way ANOVAs with Dunnett’s *post hoc* test and a Kruskal-Wallis test were test used when appropriate. *, *P* ≤ 0.05; **, *P* ≤ 0.01; ***, *P* ≤ 0.001 (indicates a significant difference compared to the mock-vaccinated and challenged [MVC] group).

BALB/c mice were immunized and boosted 3 weeks after the priming vaccination. On day 35 after the initial vaccination, the mice were challenged with the B. pertussis strain UT25Sm1. The respiratory bacterial burden was determined at 1, 3, and 14 days postchallenge (Fig. 1). At day 1 postchallenge, the bacterial burdens from both the trachea and nasal lavage fluid, but not the lung, were significantly decreased compared to the mock-vaccinated and challenged (MVC) mice for all groups (Fig. 1B and C). At day 3 postchallenge, only the curdlan-adjuvanted aP vaccine did not result in a significant decrease in the lung bacterial burden compared to the MVC mice. At day 14 postchallenge, all i.n. vaccinated groups as well as the convalescent group had decreased bacterial burdens in the lung compared to the MVC group (Fig. 1D and G). In the nasal lavage fluid, a decrease in the bacterial burden was observed compared to MVC at day 3 postchallenge for all vaccine groups and the convalescent group (Fig. 1F). In the trachea, all but the nonadjuvanted aP vaccine resulted in a significant decrease in the bacterial burden at day 3 postchallenge (Fig. 1E). Overall, these data demonstrate that when BALB/c mice are challenged with B. pertussis, i.n. vaccination is able to protect mice and reduce the bacterial burden throughout the respiratory tract to levels similar to those in convalescent mice in our 35-day study.

### Intranasal immunization induces production of B. pertussis- and antigen-specific IgG in the serum and lung supernatants and anti-B. pertussis IgA in the nasal lavage fluid and lung in the short-term study.

**FIG 2:**
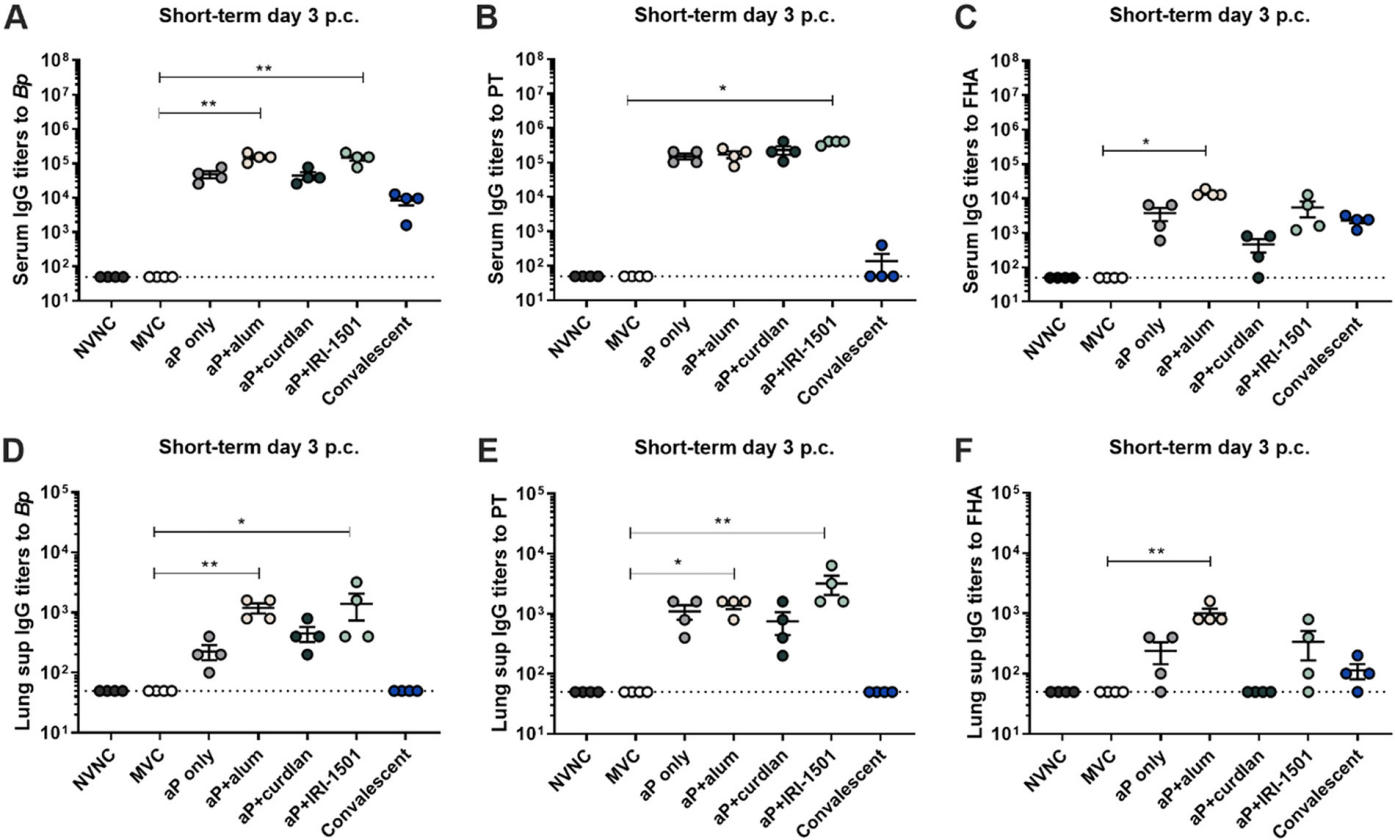
Short-term IgG antibody titers (log_10_) to B. pertussis (*Bp*) and vaccine antigens (PT and FHA) in serum (A to C) and lung supernatants (Lung sup) (D to F) 3 days after challenge were determined by an ELISA. (A to C) Serum IgG antibody titers to B. pertussis (A), PT (B), and FHA (C) were determined for all groups at 3 days postchallenge (p.c.). (D to F) Lung supernatant IgG titers to B. pertussis (D), PT (E), and FHA (F) at 3 days postchallenge. Data are presented as means ± SEM (*n* = 4 per treatment group). A Kruskal-Wallis nonparametric test with Dunnett’s *post hoc* test was used. The dotted line indicates the lowest limit of detection. *, *P* ≤ 0.05; **, *P* ≤ 0.01 (indicates a significant difference from the mock-vaccinated and challenged [MVC] group). NVNC, nonvaccinated/nonchallenged.

**FIG 3:**
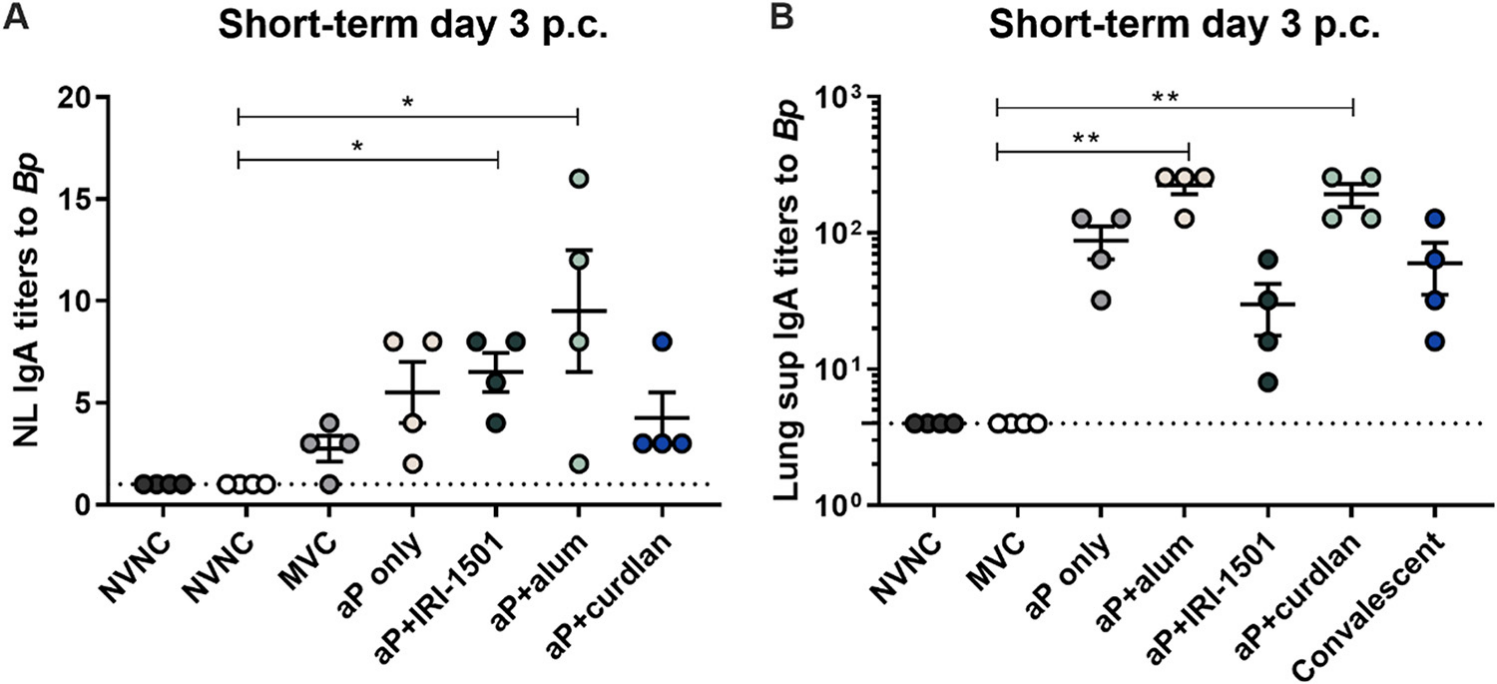
Nasal lavage fluid and lung supernatant (Lung sup) anti-B. pertussis IgA antibodies measured at 3 days postchallenge (p.c.) by an ELISA for the short-term study. (A) Nasal lavage fluid anti-B. pertussis IgA antibody titers at 3 days postchallenge. (B) Pulmonary anti-B. pertussis IgA titers at 3 days postchallenge. Results are shown as means ± SEM (*n *= 4). *, *P* < 0.05; **, *P* < 0.01. *P* values were determined by a Kruskal-Wallis test with Dunnett’s *post hoc* test. The dotted line indicates the lowest limit of detection. NVNC, nonvaccinated/nonchallenged; MVC, mock vaccinated and challenged.

We hypothesized that i.n. immunization would elicit strong local and systemic antibody responses and that these responses would vary between the different vaccine formulations. To test this hypothesis, enzyme-linked immunosorbent assays (ELISAs) were performed to detect antibodies to B. pertussis, PT, and FHA within the serum, lung supernatant, and nasal lavage fluid. Serum and lung supernatant anti-B. pertussis IgG titers were elevated for all vaccine candidates and the convalescent group at day 3 postchallenge but were statistically significant only for IRI-1501- and alum-adjuvanted aP vaccines (Fig. 2A and D). At day 3 postchallenge, anti-PT IgG titers were significant for IRI-1501-adjuvanted vaccines compared to MVC in the serum, and both IRI-1501- and alum-adjuvanted aP vaccines induced elevated anti-PT titers in the lung compared to MVC, similar to anti-B. pertussis IgG titers (Fig. 2B and E). Interestingly, anti-PT IgG titers were not observed in the convalescent group. Titers against the bacterial adhesion protein FHA suggested that only the alum-adjuvanted aP vaccine induced significant anti-FHA antibody titers in both the lung and serum (Fig. 2C and F). In addition to serum and pulmonary titers, we were also interested in evaluating IgA titers in both the nasal lavage fluid and lung in order to determine if there had been induction of a local mucosal immune response. IgA antibodies to B. pertussis are typically detected following natural infection but not after intramuscular DTP or DTaP vaccination (9). We observed that i.n. vaccinations were able to elevate IgA titers in the nasal lavage fluid and lung (Fig. 3). Compared to MVC at day 3 postchallenge, IRI-1501- and curdlan-adjuvanted aP vaccines resulted in significant B. pertussis-specific IgA titers in the nasal lavage fluid as well as pulmonary anti-B. pertussis IgA for IRI-1501-adjuvanted and alum-adjuvanted aP mice (Fig. 3A and B).

### Pulmonary proinflammatory cytokines were reduced in intranasal vaccination groups after challenge in the short-term study.

**FIG 4:**
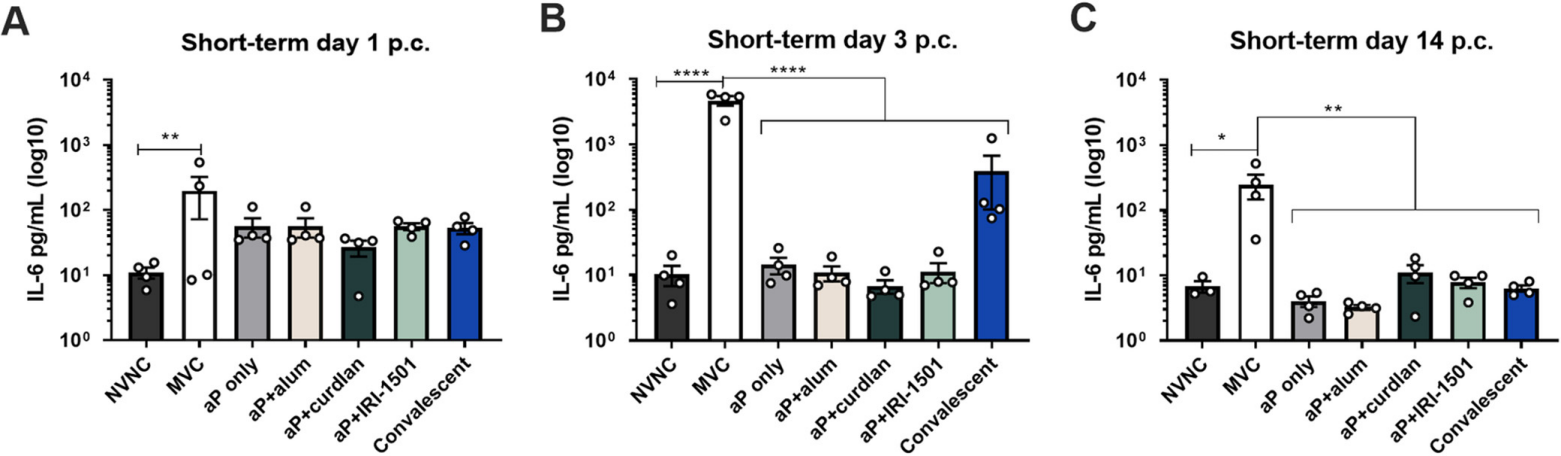
Proinflammatory cytokine IL-6 detected in the lung supernatant at 1, 3, and 14 days postchallenge (p.c.) for the short-term study using Magpix multiplex cytokine analysis. IL-6 was analyzed in the lung supernatant (Lung sup) at 1 day (A), 3 days (B), and 14 days (C) postchallenge. Results are shown as means ± SEM (*n *= 4). *, *P* ≤ 0.05; **, *P* ≤ 0.01; ****, *P* ≤ 0.0001. *P* values were determined by a Kruskal-Wallis test with Dunnett’s *post hoc* test. NVNC, nonvaccinated/nonchallenged; MVC, mock vaccinated and challenged.

IL-6, a proinflammatory cytokine, plays an important role in the adaptive immune response as it can induce Th17 polarization (36). IL-6 is elevated in the lungs during active murine B. pertussis infection (20, 36, 37). We hypothesized that i.n. administration of the experimental vaccines would result in decreased levels of proinflammatory cytokines after challenge but that the MVC mice would have increased proinflammatory cytokines due to increased bacterial burdens. For the short-term study, cytokine concentrations were determined from the lung homogenate supernatant at days 1, 3, and 14 postchallenge. We observed significant IL-6 production in the lungs of both the MVC and convalescent mice at day 3 postchallenge, but IL-6 levels for all i.n. vaccinated mice were not statistically different from those of the nonvaccinated/nonchallenged (NVNC) group (Fig. 4; see also Fig. S3 in the supplemental material). Cytokine data suggest that the i.n. vaccines prevented the need for a highly inflammatory environment in order to clear the bacteria. Previous studies indicate that convalescent animals, when rechallenged with B. pertussis, have high serum levels of IL-17 (7, 38). We also observed similar results in both the short- and long-term studies, and the IL-17 levels in the lung supernatant of the convalescent group were significant (data not shown). When looking at IL-17 production in the lungs for vaccinated mice compared to MVC mice, the combination of aP and IRI-1501 (aP+IRI-1501) showed significantly elevated IL-17 (Fig. S4).

### IgG serum titers from intranasally vaccinated mice remained elevated for the duration of the long-term study and correlated with decreased bacterial burdens in the respiratory tract of intranasally immunized mice.

**FIG 5:**
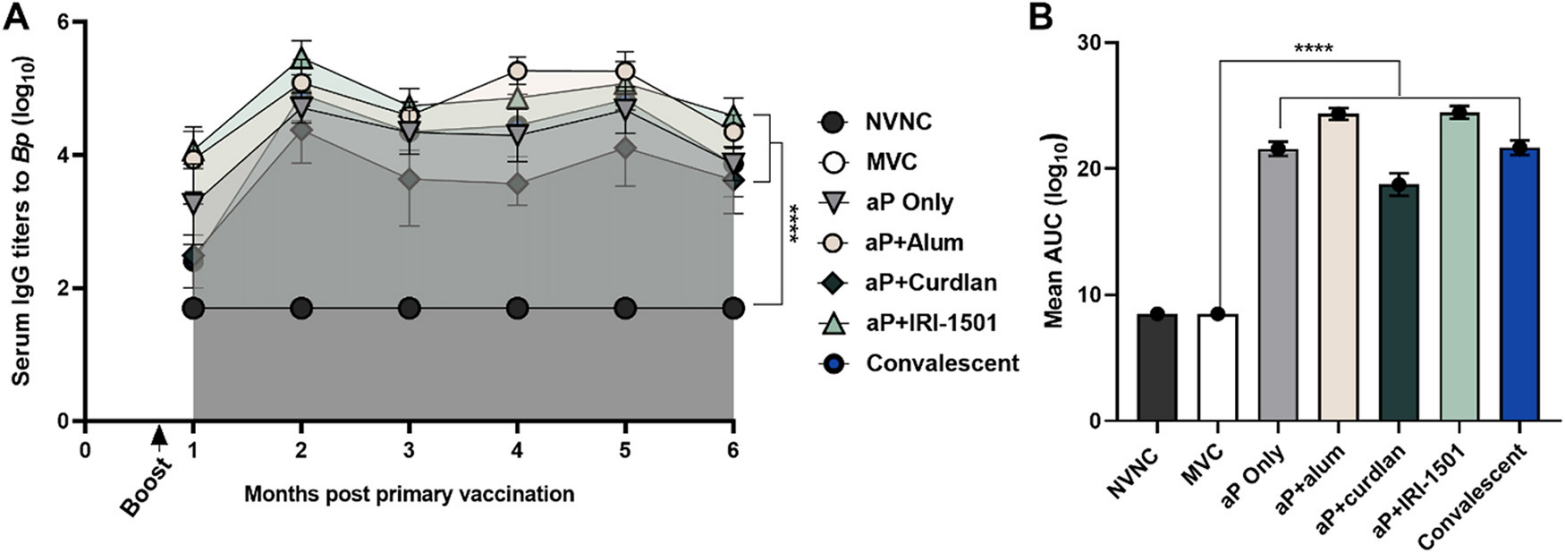
In the long-term study, IgG titers to B. pertussis were detected each month after vaccination by an ELISA, and area under the curve (AUC) analysis was performed on the data set. Shown are AUCs for IgG antibody titers to B. pertussis (A) and mean AUCs for the duration of the study (B) compared to the mock-vaccinated and challenged (MVC) group. Data are presented as means ± SEM (*n* = 5 to 10 per treatment group). The asterisks refer to statistical significance compared to the MVC group. ****, *P* ≤ 0.0001; NVNC, nonvaccinated/nonchallenged; p.c., postchallenge.

The longevity of anti-B. pertussis antibodies after vaccination was of interest after observing initial protection from all i.n. vaccine candidates in the shorter prime, boost, and challenge model. We hypothesized that i.n. vaccination would induce vaccine antigen- and B. pertussis-specific IgG titers but that decreases in titers would occur over time. Our goal was to monitor IgG antibody titers to B. pertussis after vaccination out to 6 months postboost. For the duration of the study, IgG antibody titers to B. pertussis remained elevated for the i.n. vaccinated groups as well as the convalescent group (Fig. 5). Therefore, we hypothesized that we would observe protection from B. pertussis challenge at 6 months postboost.

**FIG 6:**
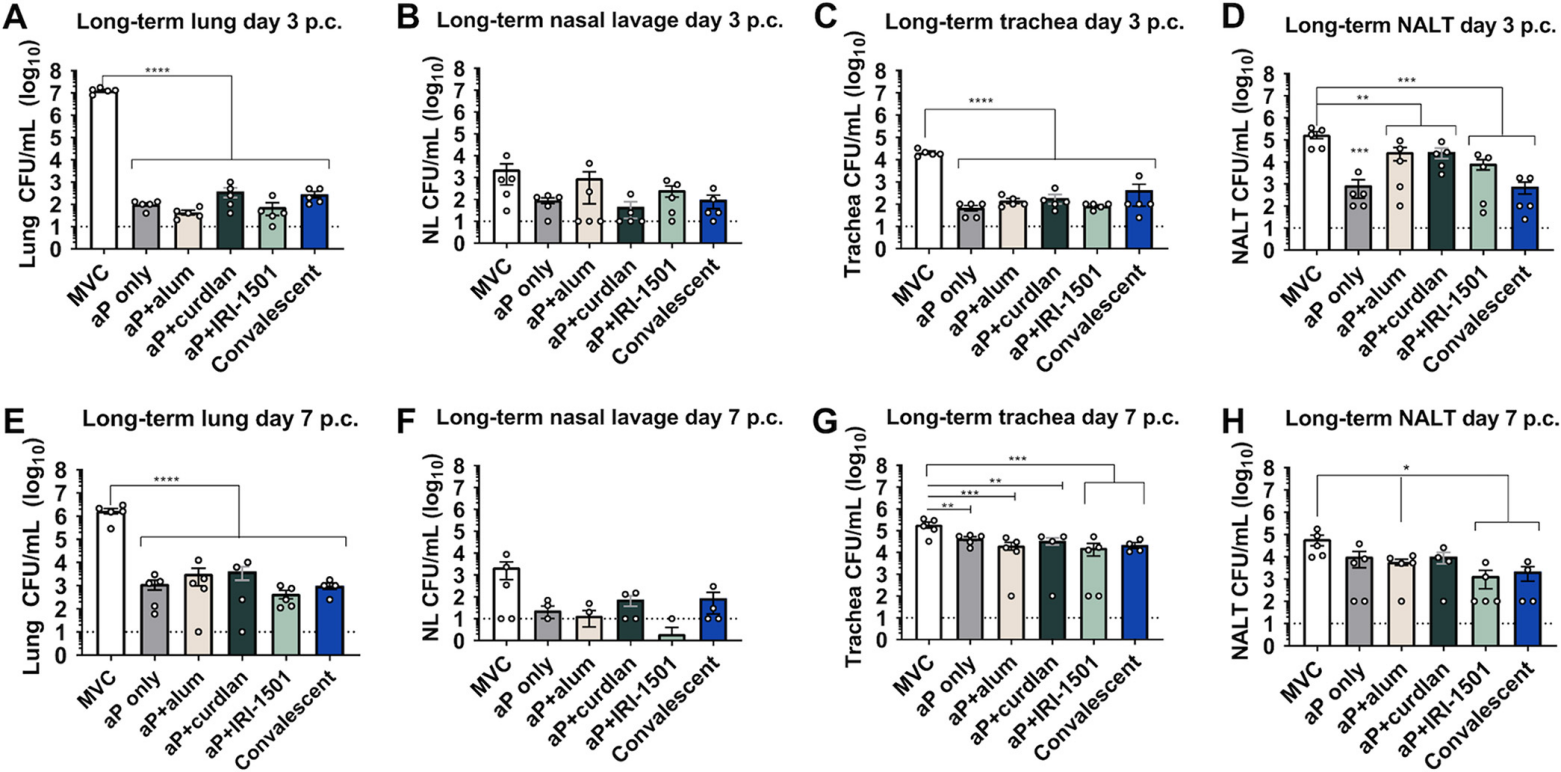
In the long-term study, the bacterial burdens in the respiratory tract (lung, trachea, nasal lavage fluid, and NALT) 3 and 7 days after B. pertussis (*Bp*) challenge were determined by CFU. Bacteria were quantified by counting serially diluted CFU following challenge. CFU counts were determined from the lung homogenate (A and E), nasal lavage fluid (B and F), trachea homogenate (C and G), and NALT (D and H). Data are presented as means ± SEM (*n* = 5 per treatment group for days 3 and 7 postchallenge [p.c.], with four averaged technical replicates). Comparisons were analyzed by one-way ANOVA with Dunnett’s *post hoc* test and a Kruskal-Wallis test, when appropriate. The dotted line indicates the lowest limit of detection. *, *P* ≤ 0.05; **, *P* ≤ 0.01; ***, *P* ≤ 0.001; ****, *P* ≤ 0.0001 (indicates significance compared to the mock-vaccinated and challenged [MVC] group). NVNC, nonvaccinated/nonchallenged.

For the long-term study, we observed protection against bacterial colonization in the respiratory tract for all i.n. vaccinated mice (Fig. 6). The B. pertussis bacterial burdens in the lung, trachea, and nasal-associated lymphoid tissue (NALT) were significantly lower for all i.n. vaccine formulations at day 3 postchallenge than for MVC, and the bacterial burden observed in the i.n. vaccination groups was not statistically different from that of the convalescent group (Fig. 6A, C, and D). At day 7 postchallenge, the bacterial burdens in the lungs and trachea for all i.n. vaccination groups and the convalescent group were also significantly reduced from MVC (Fig. 6E and G). The bacterial burdens in the NALT at 7 days postchallenge were reduced for aP+alum and aP+IRI-1501 compared to MVC but not for aP only or curdlan-adjuvanted vaccines (Fig. 6H). The fold change in the bacterial burden in the lung was more appreciable in the 7-month study than in the 35-day study, and this may be due to the fact that lower limits of detection were chosen for the lung and nasal lavage fluid after observing many groups in the shorter study below the limit of detection. Together, these long-term data suggest that i.n. vaccines can provide protection against bacterial colonization in the respiratory tract for both short- and long-term prime, boost, and challenge studies.

### Postchallenge B. pertussis-specific IgG titers in the serum and lung supernatant from the long-term study mimic the results of the short-term study, and titers negatively correlate with bacterial burdens.

**FIG 7:**
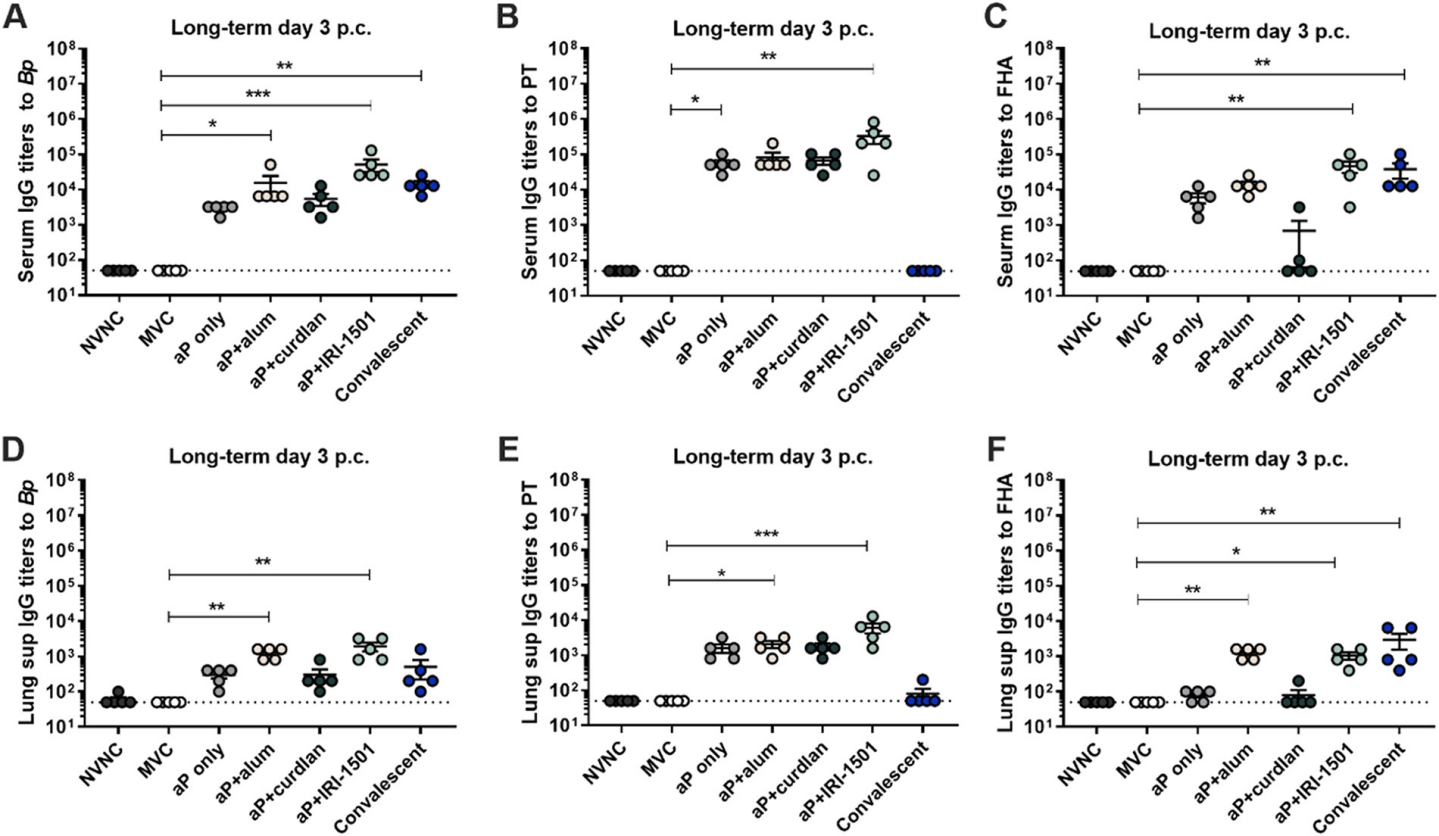
IgG antibody titers (log_10_) from the long-term study analyzed in the serum (A to C) and the lung supernatant (Lung sup) (D to F) at 3 days postchallenge (p.c.) by an ELISA. (A to C) Serum IgG antibody titers to B. pertussis (A), PT (B), and FHA (C) after challenge. (D to F) IgG titers in the lung supernatant analyzed at 3 days postchallenge for B. pertussis (D), PT (E), and FHA (F). Data are presented as means ± SEM (*n* = 5 per treatment group). Comparisons were analyzed by a Kruskal-Wallis nonparametric test with Dunnett’s *post hoc* test. The dotted line indicates the lowest limit of detection. *, *P* ≤ 0.05; **, *P* ≤ 0.01; ***, *P* ≤ 0.001 (indicates a significant difference from the mock-vaccinated and challenged [MVC] group). NVNC, nonvaccinated/nonchallenged.

In the serum, anti-B. pertussis antibodies were significantly elevated for all adjuvanted vaccines, except for curdlan at 3 days postchallenge compared to MVC (Fig. 7A). Antigens only (aP) and aP+IRI-1501 had increased anti-PT titers in the serum, but in the lung, aP+IRI-1501 along with aP+alum induced significant anti-PT IgG compared to MVC at day 3 postchallenge (Fig. 7B and E). At day 7 postchallenge, both anti-B. pertussis and anti-PT in the serum and lung were significant for aP, aP+alum, and aP+IRI-1501 compared to MVC (Fig. S5). There were significant anti-FHA and anti-B. pertussis antibodies present in the serum and increased anti-FHA in the lung at day 3 postchallenge for the convalescent group compared to MVC (Fig. 7A, C, and F). While not significant, several mice in the convalescent group had detectable anti-PT antibodies at 7 days postchallenge (Fig. S5). Th1 (IgG2a and IgG2b) and Th2 (IgG1) antibodies recognizing B. pertussis were observed in the serum at 7 days postchallenge. The ratio of Th1/Th2 was higher for convalescent mice than for i.n. vaccinated mice; however, significant IgG2a titers were also observed after i.n. vaccination (Fig. S7).

The production of IgA antibodies was significantly induced in the nasal lavage fluid in the short-term study but not the long-term study, indicating that anti-B. pertussis IgA may be a short-term mucosal immune response (Fig. 3 and Fig. S6). The levels of anti-B. pertussis IgA in the lung were also significant compared to MVC only for the alum-adjuvanted aP vaccine group (Fig. S6). Overall, these data suggest that IgG antibodies specific to both B. pertussis and its toxins are still being produced 6 months after the boost.

### Levels of IL-6 and other proinflammatory cytokines were reduced in the intranasal vaccination groups after challenge in the long-term study.

**FIG 8:**
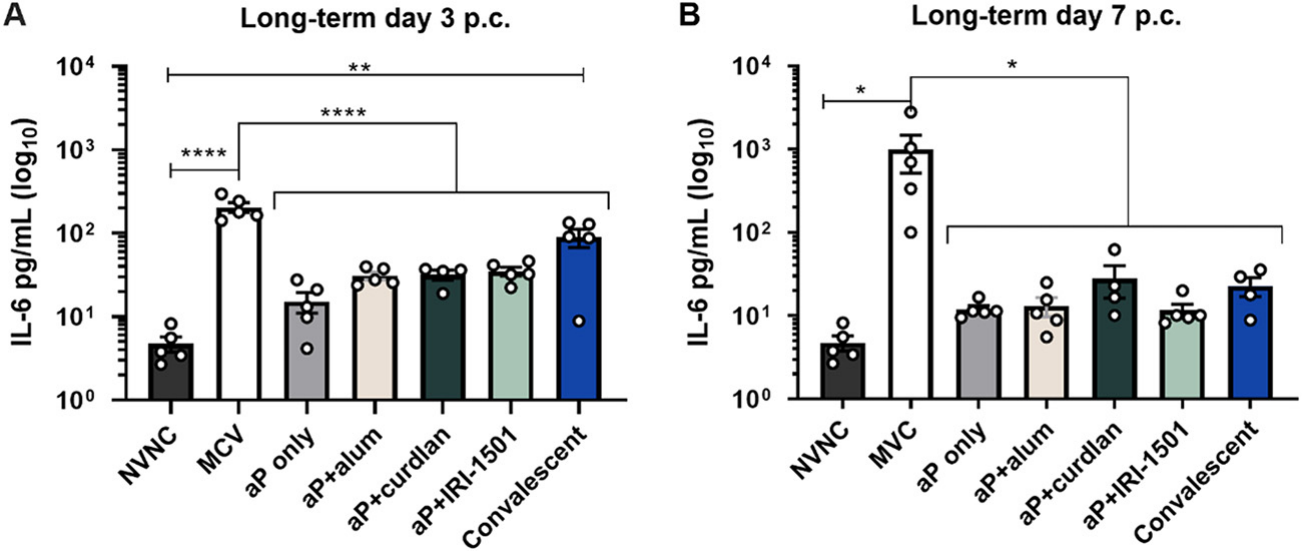
Pulmonary proinflammatory cytokine IL-6 assessed at 3 and 7 days postchallenge (p.c.) for the long-term study using Magpix multiplex cytokine analysis. Pulmonary IL-6 production at 3 days (A) and 7 days (B) postchallenge was assessed. Results are shown as means ± SEM (*n *= 5). *, *P* < 0.05; **, *P* < 0.01; ****, *P* < 0.0001. *P* values were determined by one-way ANOVA with Dunnett’s *post hoc* test compared to mock-vaccinated and challenged (MVC) mice. NVNC, nonvaccinated/nonchallenged.

For the long-term study, the lung homogenate supernatant was collected at days 3 and 7 postchallenge for cytokine analysis. Long-term cytokine production profiles after challenge were similar to those in the short-term study (Fig. S3). As in the short-term study, IL-6 production in the lungs for MVC mice was significant compared to the vaccinated groups and controls (NVNC and convalescent) (Fig. 8). Additionally, heat maps of proinflammatory cytokines in the lung for both the short- and long-term studies indicate that the MVC and convalescent groups had induction of proinflammatory cytokines (IL-18, tumor necrosis factor alpha [TNF-α], and interferon gamma [IFN-γ]) (Fig. S3).

### Intranasal vaccination alters pulmonary T cells after challenge in the long-term study.

**FIG 9:**
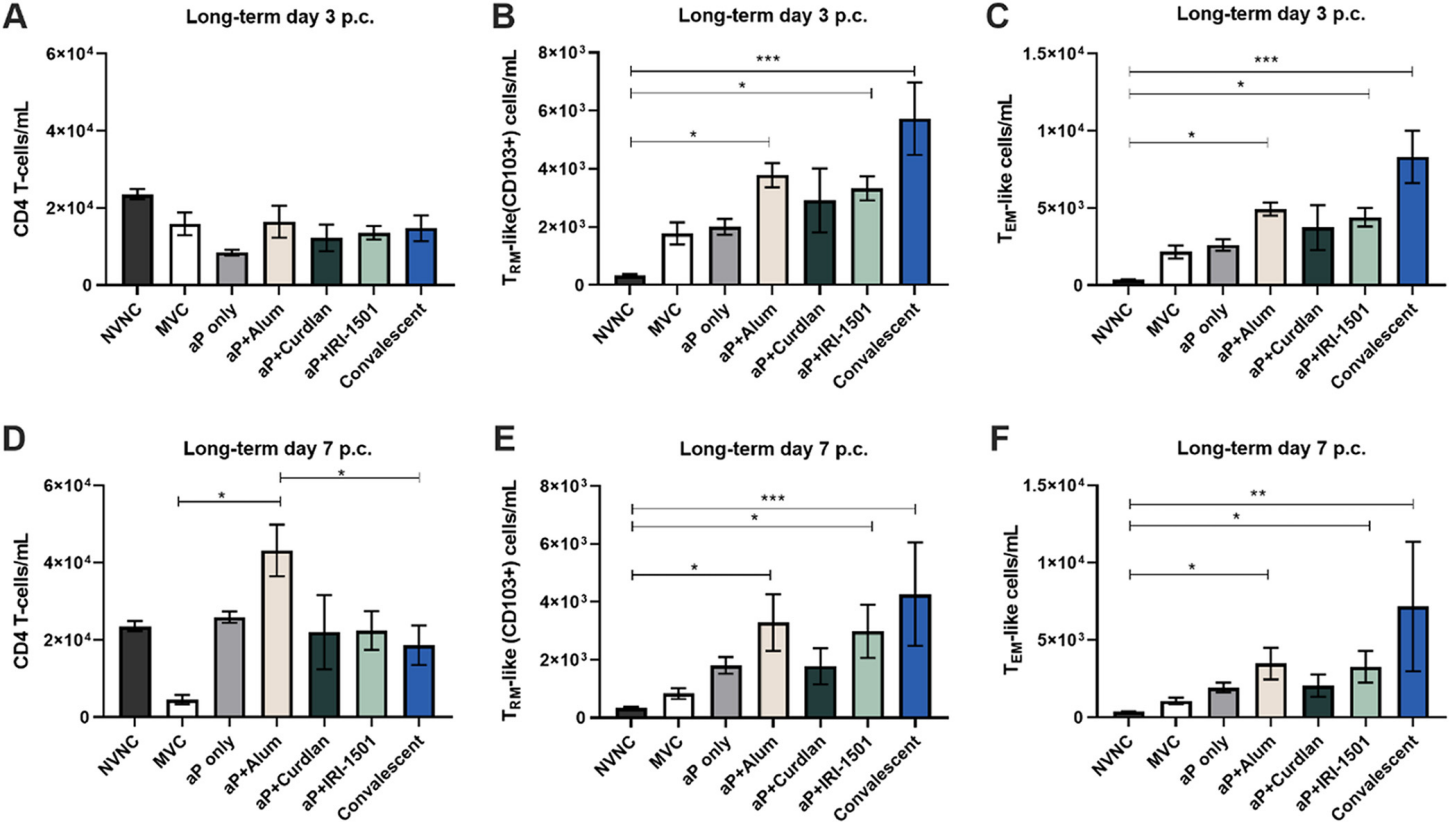
In the long-term study, pulmonary T-cell populations were detected via flow cytometry at 3 and 7 days postchallenge (p.c.). (A and D) CD4^+^ T-cell populations at 3 days (A) and 7 days (D) postchallenge. (B and E) Proportions of tissue-resident memory-like T (T_RM_) cells at 3 days (B) and 7 days (E) postchallenge. (C and F) Tissue effector memory-like T (T_EM_) cells at 3 days (C) and 7 days (F) postchallenge. Data are presented as mean values ± SEM (*n* = 5 per treatment group). Comparisons were analyzed by ANOVA followed by Tukey’s multiple-comparison test. The asterisks refer to statistical significance compared to the nonvaccinated/nonchallenged (NVNC) or mock-vaccinated and challenged (MVC) group. *, *P* ≤ 0.05; **, *P* ≤ 0.01; ***, *P* ≤ 0.001.

Previous studies report that natural infection with B. pertussis significantly increases the amount of tissue-resident memory (T_RM_) cells in the lung, and additional studies indicate that WCV, but not acellular vaccines, increases T_RM_ cells (39). We hypothesized that i.n. vaccination with our selected adjuvants would increase the T_RM_ cell population in the long-term study. Therefore, we examined the presence of T cells in the lung at 6 months postboost. We observed that T_RM_-like (CD4^+^ CD44^+^ CD62L^−^ CD103^+^) cells were significantly elevated for the convalescent group, as well as the alum- and IRI-1501-adjuvanted aP vaccine groups, compared to NVNC mice (Fig. 9B and E). In addition to T_RM_ cells, we investigated the presence of T effector memory (T_EM_)-like cells, which were CD4^+^ CD44^+^ CD62L^−^ (Fig. 9C and F). T_EM_ cells recirculate between lymphoid organs and tissues, and when a host has a second exposure to an antigen, these cells expand and offer additional circulating protection (40). The T_EM_-like cells were also significantly elevated for the convalescent, aP+alum, and aP+IRI-1501 groups compared to NVNC mice in the long-term study (Fig. 9C and F).

### The number of IgG-secreting cells in the bone marrow was elevated for intranasally vaccinated mice.

**FIG 10:**
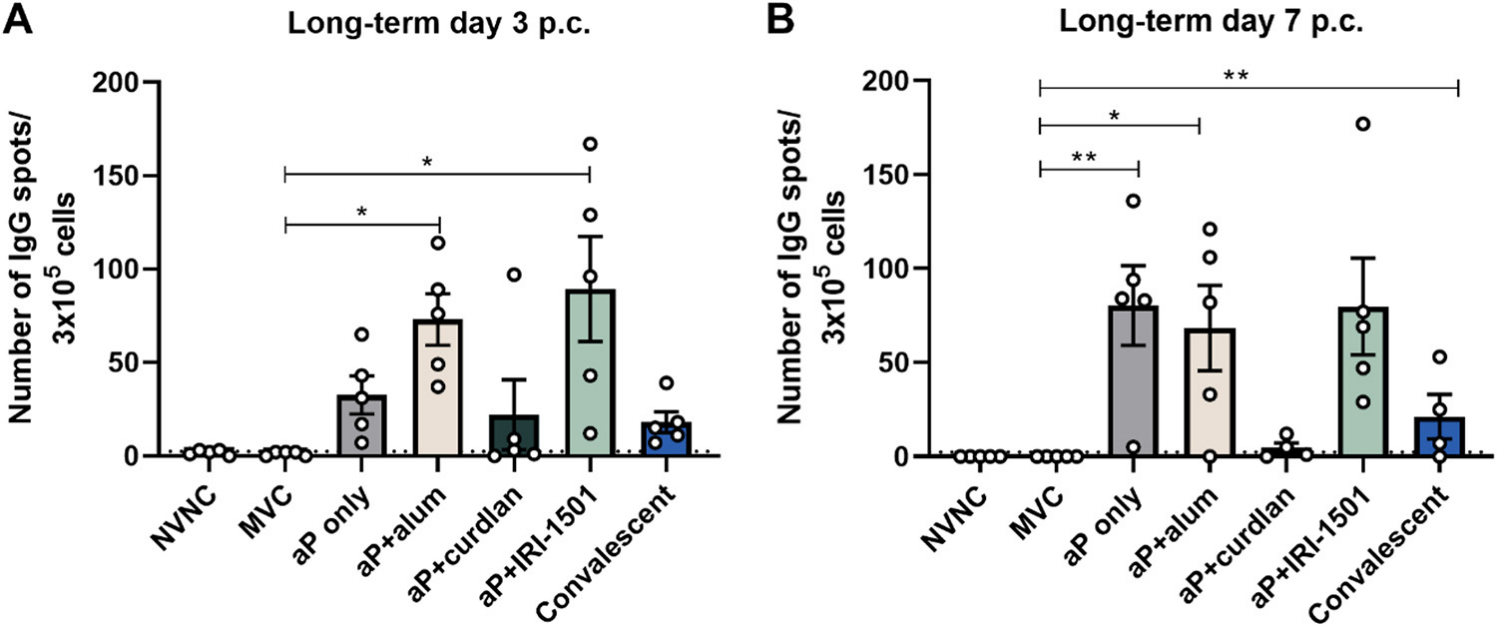
B. pertussis-specific IgG counts from cells isolated from bone marrow 3 and 7 days after B. pertussis challenge determined by an ELISpot assay for the long-term study. The numbers of B. pertussis-specific IgG-producing B cells at day 3 postchallenge (p.c.) (A) and day 7 postchallenge (B) are shown. Data are presented as means ± SEM (*n *= 5). *, *P* < 0.05; **, *P* < 0.01. *P* values were determined by one-way ANOVA with Dunnett’s *post hoc* test compared to mock-vaccinated mice for statistical analysis. NVNC, nonvaccinated/nonchallenged; MVC, mock vaccinated and challenged.

While there is no universally agreed-upon correlate of protection for B. pertussis, antigen-specific antibodies can protect against B. pertussis infection (41, 42). After antigens are taken up by follicular dendritic cells, they are presented to both T follicular helper cells and germinal center B cells, a process known as the germinal center reaction. Short-lived and long-lived plasma cells, which secrete antibodies that have a high affinity for a particular antigen, are the result of a germinal center reaction. Once created, the plasma cells migrate to the bone marrow. Therefore, we collected bone marrow cells and performed an enzyme-linked immunosorbent spot (ELISpot) analysis in order to enumerate the number of plasma cells that produced B. pertussis-specific IgG. The number of plasma cells secreting anti-B. pertussis-specific antibodies was elevated compared to MVC for alum- and IRI-1501-adjuvanted vaccines at days 3 and 7 postchallenge (Fig. 10). IgG-specific B. pertussis antibodies were also elevated in mice immunized with the nonadjuvanted (aP) vaccine on day 7 postchallenge (Fig. 10B).

### Negative correlation in the long-term study between B. pertussis titers in intranasal vaccinated groups and bacterial burdens in the lungs.

**FIG 11:**
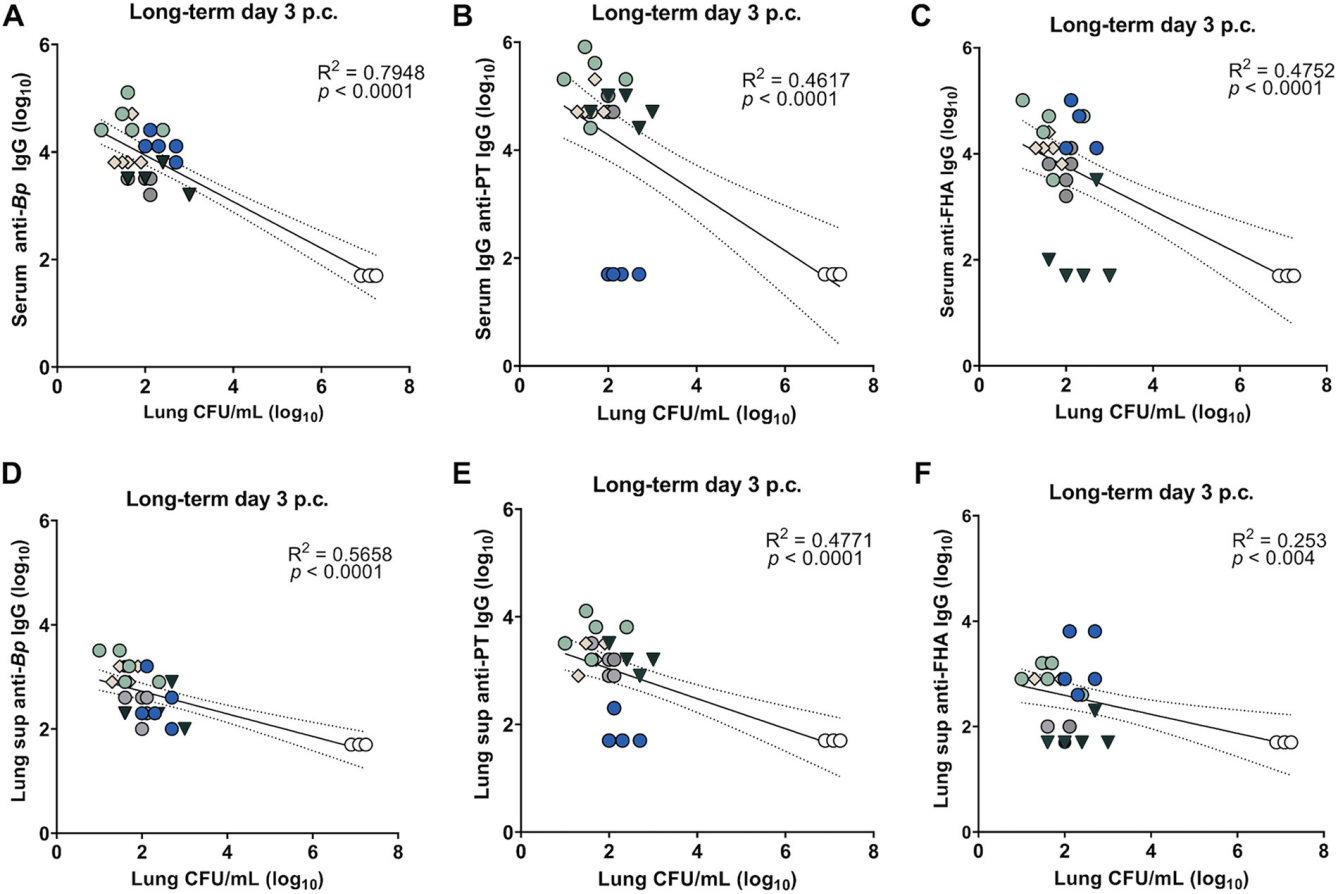
Linear regression of B. pertussis (*Bp*)- or antigen-specific serum and lung IgG titers against the bacterial burden in the lung at day 3 postchallenge (p.c.) for the long-term study. (A to C) IgG serum titers to B. pertussis (A), PT (B), and FHA (C) correlated with bacterial burdens in the lung, at the same time point. (D to F) Correlation of IgG lung supernatant titers to B. pertussis (D), PT (E), or FHA (F) at 3 days postchallenge to lung bacterial burdens. White circles, mock vaccinated and challenged (MVC); gray circles, aP only; tan diamonds, aP+alum; forest green upside-down triangles, aP+curdlan; light green circles, aP+IRI-1501; cobalt circles, convalescent group. NVNC, nonvaccinated/nonchallenged.

In humans, serological protection is not a universally agreed-upon assessment of long-term protection. However, in our murine studies, the humoral immune response correlates with a decreased bacterial burden. Correlations between bacterial burdens in the lung and the anti-B. pertussis, anti-PT, and anti-FHA IgG titers in the lung and serum for i.n. vaccinated mice were significant (Fig. 11). Correlations were also observed between IgG titers and bacterial burdens in other respiratory tissues (Fig. S8). It should be noted that the anti-PT antibody titers were low in the lungs for the convalescent group, especially compared to vaccinated mice, but the bacterial burden was also significantly reduced (Fig. 11). A negative correlation between T_RM_-like cells in the lungs of the convalescent and i.n. vaccinated groups and lung bacterial burdens was also observed (Fig. S8).

## DISCUSSION

Many countries use intramuscular acellular vaccines to protect against B. pertussis infection. However, the immunity afforded by acellular vaccine formulations is suggested to wane as much as 30% each year postvaccination, and immunized individuals may be asymptomatic carriers (4, 43). Given that B. pertussis colonizes the ciliated cells in the respiratory mucosa and consequently results in an upper respiratory infection that can sometimes disseminate to the lower respiratory tract, a mucosal vaccine that induces immunity at the site of infection may provide superior long-term protection. Mucosal vaccinations with oral and intranasal (i.n.) pertussis vaccine formulations have led to protective immune responses (12, 17, 20, 44).

One mucosal vaccination strategy currently in clinical trials focuses on i.n. immunization with BPZE1, a live attenuated B. pertussis strain. Modification of three important virulence factors (PT, tracheal cytotoxin [TCT], and dermonecrotic toxin [DNT]) results in BPZE1’s attenuation, but other key virulence factors like adenylate cyclase toxin (ACT) are still active (19, 44, 45). BPZE1 provides protection in both murine and baboon models. With regard to the murine model, BPZE1 provides both early and long-term protection and results in an increase of lung T_RM_ cells (17, 18, 46, 47). BPZE1 induces local and systemic B. pertussis-specific IgG and IgA antibodies (19). The live attenuated strain is also able to generate anti-PT IgG antibodies, but murine and clinical studies demonstrate that there is a prolonged period of time before antibody induction occurs. The delay in anti-PT IgG production can be explained by the strain needing to colonize the respiratory tract and produce the gPT antigen before antibodies to gPT can be generated (17, 18, 46, 48).

The role of humoral immunity in long-term protection is still debated, and while there is no agreed-upon long-term correlate of protection for pertussis vaccination in humans, anti-PT IgG levels of >5 IU/ml have been considered protective (49). Pertussis toxin can inhibit the recruitment of neutrophils and therefore prevent the rapid antibody-mediated clearance of B. pertussis, suggesting the importance of anti-PT antibodies in pathogen neutralization (50). In our mucosal vaccination study, we observed considerable induction of anti-PT antibodies in the serum and lung of vaccinated mice in both the short- and long-term studies but did not observe elevated anti-PT titers in the convalescent group (Fig. 2 and 7). Nguyen et al. also demonstrated that the anti-PT antibody hu1B7 can prevent leukocytosis and other clinical features of pertussis infection in a neonatal baboon model, further supporting its benefit in protection (51). Additionally, individuals who have higher anti-PT antibodies after immunization are better protected from infection and have decreased disease severity in households with B. pertussis exposure (52). Collectively, these studies indicate the importance of anti-PT antibodies, and in our study, we observed that i.n. IRI-1501- or alum-adjuvanted aP vaccines increase the humoral immune response to B. pertussis-specific antigens, including PT.

A recent publication reporting intranasal vaccination using outer membrane vesicles of pertussis (omvPV) showed the induction of anti-OMV IgG- and IgA-secreting plasma cells as well as IgG and IgA memory B cells that were specific to OMVs (15). Local (IgA) and systemic (IgG) antibodies recognizing several pertussis-specific virulence factors, including FHA, an autotransporter (Vag8), *Bordetella* resistance to killing (BrkA), and fimbria 2/3 (Fim2/3), were induced after vaccination, but anti-PT antibodies were not observed (15). Subcutaneous and i.n. vaccination with omvPV polarized a Th1/Th17 response, which is also seen with both natural infection and whole-cell vaccine administration (7, 15, 53). A substantial number of studies highlight the correlation between a Th1/Th17 immune response and long-term protection (7, 14, 39). We selected β-glucans as adjuvants to combine with the aP vaccine as studies suggest that they increase IL-17a and induce Th17 polarization (10, 29). Previously, we observed a slight increase in IL-17a after i.n. vaccination with curdlan-adjuvanted DTaP in CD-1 mice (20). In the current study, we observed a Th1/Th17 response in our convalescent group, but our i.n. vaccines did not appear to induce robust Th1/Th17 polarization. However, we again observed increases in pulmonary IL-17a and the Th17 immune response after immunization with alum or the β-glucan IRI-1501 (Fig. S5).

In a previous study, we assessed T_RM_-like cells in the lungs of CD-1 mice after i.n. vaccination with DTaP and DTaP adjuvanted with curdlan. In this study. we observed (while not significant) a slight increase of the T_RM_-like cell population, prompting us to continue investigating these cells in our long-term study using BALB/c mice (20). In our long-term study, we observed increases in T_EM_- and T_RM_-like cells in the lung for our alum- and IRI-1501-adjuvanted aP groups (Fig. 9B and C). Wilk et al. demonstrated the importance of T_RM_ cells in the longevity of B. pertussis protection and that blocking T_RM_ cells prevented bacterial clearance in immunized mice (39). Another study by Allen et al. focused on the use of an acellular i.n. vaccine combined with LP-GMP (a combination of an intracellular receptor stimulator of interferon gene [STING] agonist and a ligand of Toll-like receptor 2 [TLR2]) and observed that the inclusion of LP-GMP with the aP vaccine induced significant IL-17-producing T_RM_ cells and provided long-term protection (10 months) against B. pertussis aerosolization challenge (14). Their novel adjuvant provided superior protection over their alum-adjuvanted acellular vaccine in the nasal cavity. Our alum-adjuvanted vaccine provided long-term protection (7 months) in the nasal cavity; however, we used higher doses of vaccine antigens than those in the acellular formulation used by Allen et al. and intranasal administration of B. pertussis compared to an aerosolization challenge model, which could be responsible for the differences between these studies’ findings (14).

In summary, in this i.n. study, the novel β-glucan IRI-1501 and alum were superior adjuvants compared to curdlan or an unadjuvanted vaccine. Both vaccine formulations reduced bacterial burdens, induced robust antibody responses in the short- and long-term studies, and prompted additional protective immune responses such as antigen-specific IgG-secreting plasma cells in the bone marrow and T_RM_- and T_EM_-like cells in the lung. Alum is a well-studied and currently approved adjuvant for DTaP, making it advantageous to include it in an experimental i.n. vaccine. Alum acts like a mild irritant that can recruit leukocytes and skew immunity toward a Th2 immune response, which is less effective at protecting from a B. pertussis infection (27, 28, 54). IRI-1501, on the other hand, is a novel whole β-glucan particle (WGP) adjuvant. WGP adjuvants are acid resistant, making them ideal for a mucosal vaccine, and are efficient at antigen loading and delivery to antigen-presenting cells (29). In our study, IRI-1501 was capable of providing both local and systemic humoral immune responses, increasing antigen-specific plasma B cells, inducing pulmonary T_RM_ cells, and reducing lung proinflammatory cytokines while also inducing pulmonary IL-17a production (Fig. 2 to 4 and Fig. 7 to 10).

While additional work is still needed, we propose that IRI-1501 is worth exploring as a novel i.n. adjuvant. In future studies, aP antigens will be loaded or cross-linked with fluorescently labeled IRI-1501, allowing us to track the vaccine’s localization in the respiratory tract, similar to our previous i.n. vaccination study using β-glucans (20). We also plan to combine our vaccine with the RTX (repeats in toxin domain of ACT) antigen to investigate if RTX’s addition can increase protection, as we previously observed in an intraperitoneal DTaP+RTX vaccination study (37). Preclinical, nonhuman primate, and clinical studies are still needed to establish a superior next-generation acellular pertussis vaccine, and collectively, the data from this study support the potential of a mucosal vaccine to protect against B. pertussis.

## MATERIALS AND METHODS

### Vaccine composition.

Vaccine antigens (1.25 μg of genetically detoxified pertussis toxin [gPT] [catalog number 184; List Biologicals], 1.25 μg of filamentous hemagglutinin [FHA] [Enzo Life Sciences], and 0.8 μg of pertactin [PRN] [catalog number 187; List Biologicals]) were diluted with endotoxin-free Dulbecco’s phosphate-buffered saline (PBS) (Millipore Sigma) to make the base vaccine (aP). The mock vaccine (MV) for this study was the vaccine diluent endotoxin-free Dulbecco’s PBS (Millipore Sigma). The endotoxin content of the antigens was measured by the *Limulus* amebocyte test (Pierce *Limulus* amebocyte lysate [LAL] chromogenic endotoxin quantification kit), and small amounts of endotoxin were observed: 0.0025 endotoxin units (EU)/dose gPT, 0.00125 EU/dose FHA, and 0.0015 EU/dose PRN. Curdlan (catalog number tlrl-curd; InvivoGen) was prepared by dissolving 50 mg in 2.5 ml sterile purified water. Curdlan was brought into solution by adding 100 μl 1 N NaOH and vortexing. The curdlan suspension (20 mg/ml) was then sonicated for 10 min and placed in a 37°C water bath until administration. The curdlan adjuvant was combined with the base vaccine and administered at 200 μg per mouse. Alum alhydrogel (InvivoGen) was combined with the base vaccine at 1/20 the human DTaP dose (31.25 μg alum per vaccination) and then mixed using end-over-end rotation. IRI-1501 (Immuno Research, Inc.) was prepared with the base vaccine using 100 μg per administration (see Fig. S1 in the supplemental material). The vaccines administered in the study were prepared no longer than 1 h before administration.

### B. pertussis strains and growth conditions.

B. pertussis strain UT25Sm1 was used for murine challenge in all experiments (55). UT25Sm1 was cultured on Bordet-Gengou agar (BG; Remel) plus 15% defibrinated sheep’s blood (Hemostat Laboratories) with streptomycin at 100 μg/ml. B. pertussis was incubated at 36°C for 48 h and then transferred to modified Stainer-Scholte liquid medium (56). Liquid cultures were incubated for 24 h at 36°C, with shaking at 180 rpm, until reaching an optical density at 600 nm (OD_600_) of ∼0.6, at which time cultures were diluted for the challenge dose.

### Intranasal vaccine administration.

BALB/c (inbred; strain code 028) mice aged 4 weeks were obtained from Charles River Laboratories. At 5 weeks, the mice were anesthetized with 77 mg/kg of body weight of ketamine and 7.7 mg/kg of xylazine. Mice were administered 50 μl of the vaccine or the vehicle control intranasally (i.n.), with 25 μl in each nostril. Mice were boosted with the same vaccine formulations 21 days after priming. The convalescent group was inoculated with 20 μl of the challenge dose (2 × 10^7^ CFU), with 10 μl in each nostril on the prime day, and were not rechallenged until the completion of each study. All murine infection experiments were performed according to protocols approved by the West Virginia University Animal Care and Use Committee (protocol number 1602000797).

### Short-term challenge model.

Thirty-five days after the initial vaccination, mice were challenged with 2 × 10^7^ CFU of B. pertussis (10 μl per nostril) (Fig. S2). At days 1, 3, and 14 postchallenge, mice were euthanized, and blood was collected by cardiac puncture. White blood cell counts were determined using a Drew Scientific Hemavet, and serum was separated by centrifugation through a BD Microtainer blood collector and stored at −80°C until analysis. The trachea and lungs were removed and homogenized separately. Lungs were suspended in 2 ml of sterile PBS in gentleMACS C tubes (Miltenyi) using a GentleMACS Octo dissociator with heaters (Miltenyi), using the m_lung_02 setting. The lung homogenate was used to determine CFU via serial dilutions. A sample of the lung homogenate was used for flow cytometry, the remaining sample was centrifuged at 14,000 × *g* for 4 min, and supernatants were stored at −80°C until cytokine and antibody analyses. The trachea was homogenized in 1 ml of sterile PBS with a Polytron homogenizer. To determine the amount of B. pertussis in the nares, 1 ml of PBS was flushed through the nares and collected. The bacterial burden was determined in the lung, trachea, and nasal lavage fluid by CFU using serial dilutions. Serial dilutions were done in PBS and then plated onto BG containing streptomycin (100 μg/ml) to ensure that only B. pertussis UT25 was cultured.

### Long-term challenge model.

Mice were primed, boosted, and challenged as described above, but the challenge was performed 6 months after the boosting vaccination (Fig. S2). The bacterial burden in tissues was determined via the protocol described above in the short-term study, but mice were euthanized at 3 and 7 days postchallenge. All mice were administered 3 μg of anti-CD45-PECF594 (catalog number 562420; BD Biosciences) 5 min prior to euthanasia via the tail vein. This technique was modified from the ones described previously by Wilk et al. and Anderson et al. to expose circulating lymphocytes to anti-CD45 (39, 57). We also assessed the bacterial burden in the nasal-associated lymphoid tissue (NALT) for this study. NALT was isolated from the palate with a scalpel and homogenized in 1 ml of PBS with a Polytron homogenizer. Lungs were suspended in 1 ml of sterile PBS in a C tube and processed as described above. One hundred microliters of the lung homogenate was used to determine CFU via serial dilutions, and an additional 100 μl was spread onto a single plate to decrease the limit of detection to 10 CFU/ml. The remaining homogenate was centrifuged at 14,000 × *g* for 4 min, and supernatants were stored at −80°C until cytokine and antibody analyses. Serial dilutions for all trachea, nasal lavage fluid, and NALT samples were performed as described above; however, we spread 100 μl of the nasal lavage fluid on a BG plate in order to decrease the CFU limit of detection.

### Serological analysis of B. pertussis-specific antibodies.

Serological responses specific to B. pertussis antigens were quantified by an enzyme-linked immunosorbent assay (ELISA). High-binding microtiter plates were coated with PT (50 ng/well) (catalog number 180; List Biologicals) or FHA (50 ng/well) (Enzo Life Sciences), as described previously by Boehm et al. (37) For serological responses to B. pertussis, UT25Sm1 was cultured to an OD_600_ of ∼0.6 and diluted down to an OD_600_ of 0.245, and microtiter plates were coated with 50 μl of bacteria per well. After coating, the plates were washed three times with PBS plus 0.05% (vol/vol) Tween 20 (Fisher Scientific) (PBS-T) and blocked with 5% nonfat dry (NFD) milk in PBS-T overnight at 4°C. Blocked plates were washed with PBS-T, and serum (1:50) or lung supernatant (1:50) samples were then prepared in 5% NFD milk in PBS-T. The nasal lavage fluid was added directly to the first row of wells. All samples were serially diluted (1:2). After 2 h of incubation at 37°C, plates were washed and incubated with goat anti-mouse IgG and IgA alkaline phosphatase-conjugated antibodies (SouthernBiotech) (1:2,000) for 1 h at 37°C. Plates were then washed and incubated with Pierce *p*-nitrophenyl phosphate (PNPP) (Thermo Fisher Scientific) according to the manufacturer’s instructions for 30 min. The absorbance of the plates was read at the OD_405_ using a Synergy H1 plate reader (BioTek). Positive antibody titers were determined as any values above the baseline (set at two times the average of the blanks).

### Serological analysis over time.

Blood samples were collected from the submandibular vein of each mouse every 30 days after the initial vaccination. The samples were left at ambient temperature for 30 min to allow the blood to coagulate and then centrifuged for 2 min at 14,000 × *g*. Serum was collected and stored at −80°C until analyses were performed.

### Flow cytometry sample preparation and analysis.

The lung homogenate from the long-term study (400 μl) was diluted with 5 ml of fluorescence-activated cell sorter (FACS) buffer (PBS with 1% [vol/vol] fetal bovine serum [FBS] and 0.5% [vol/vol] EDTA), and the suspension was filtered using a 100-μm cell strainer (VWR International). Samples were centrifuged at 1,000 × *g* for 5 min, and the supernatants were discarded. Red blood cells were lysed using 1× BD Pharm Lyse for 2 min at 37°C, washed with RPMI 1640 with 10% (vol/vol) FBS, and centrifuged at 1,000 × *g* for 5 min. The pellets were resuspended in FACS buffer with Fc receptor blocker (BD Pharmingen) and incubated on ice for 15 min. Lung cells were stained with myeloid and resident memory cell surface markers (Table S1). Each cell suspension sample was incubated with the antibody cocktails corresponding to myeloid or resident memory surface markers for 1 h at 4°C in the dark. Cell suspension samples were washed with PBS, and the pellet was resuspended in 0.4% paraformaldehyde and stored overnight at 4°C in the dark. Pellets were washed with PBS and resuspended with 300 to 500 μl PBS. Samples were evaluated using an LSR Fortessa flow cytometer (BD Biosciences) and analyzed using FlowJo v10 (FlowJo, LLC). Cell counting beads (Sperotech) were used to aid in sample analysis. The flow cytometry gating strategy for myeloid cells was reported previously by Boehm et al. (20). The T-cell flow cytometry gating panel was adapted from those described previously by Wilk et al. (2019) and Sen-Kilic et al. (39, 58).

### Lung homogenate cytokine analysis.

To quantify inflammatory cytokines at the site of infection, lung homogenate supernatants were prepared, as suggested by the kit manufacturer, and diluted 1:2. Quantitative analysis of cytokines was performed using the Invitrogen Th1/Th2/Th9/Th17/Th22/Treg cytokine 17-plex ProcartaPlex panel (catalog number EPX170-26087-901), according to the manufacturer’s instructions, and samples were run on a Magpix (Luminex) instrument. Bead counts below 35 were invalidated.

### ELISpot sample preparation and analysis.

ELISpot assays were performed using a mouse IgA/IgG double-color ELISpot assay (ImmunoSpot) to enumerate the number of B cells that produced IgA and IgG antibodies specific to B. pertussis. UT25Sm1 was cultured and diluted as described above for the ELISA protocol. Polyvinylidene difluoride (PVDF) membrane 96-well plates were coated with UT25Sm1 and incubated overnight at 4°C. Bone marrow cells were isolated from long-term study groups after challenge. Both hind femurs from each mouse were removed and placed into tubes containing Dulbecco’s modified Eagle’s medium (DMEM) (Gibco). The tubes were spun at 1,000 × *g* for 2 min, and the resulting pellet was resuspended in heat-inactivated (HI), filter-sterilized FBS (Gemini Bio Products). The cells were then filtered through a 70-μm filter and placed in a solution of HI FBS plus 10% (vol/vol) dimethyl sulfoxide (DMSO) and stored at −80°C until the assay was performed. To prepare the cells for the assay, they were first thawed in a 37°C water bath and transferred into a solution of RPMI 1640 (Gibco) plus 10% (vol/vol) HI FBS. The cells were then centrifuged at 350 × *g* for 6 min, resuspended in CTL test B culture medium (ImmunoSpot), and counted. After coating, the plate was washed with PBS prior to adding cells. Four serial dilutions were used for each sample (6.25 × 10^6^, 1.25 × 10^6^, 3.13 × 10^5^, and 1.56 × 10^5^ cells/well). Once cells were added, the plate was left to incubate at 37°C overnight. ELISpot plates were imaged and counted with the ImmunoSpot S6 Entry analyzer and CTL software. The counts were analyzed, and a dilution was selected that had spot counts in the range of 10 to 100 per well.

### Statistical analysis.

Statistical analyses were performed using Prism version 10 software (GraphPad). Comparisons among three or more groups were analyzed by one-way analysis of variance (ANOVA) followed by Tukey’s multiple-comparison test for data that follow a normal distribution. Statistical analysis for nonparametric data was performed using the Kruskal-Wallis test with Dunnett’s *post hoc* test. Correlations between two parameters were calculated via linear regression analysis of log-transformed data. Differences between data sets over time were calculated by area under the curve (AUC) analysis.

### Ethics statement.

Animal work in this study was carried out in strict accordance with the recommendations in the *Guide for the Care and Use of Laboratory Animals* of the National Institutes of Health (59). The West Virginia University Institutional Animal Care and Use Committee (IACUC) approved the protocols and this research under the IACUC protocol 1602000797.

### Data availability.

Data for all figures are available upon reasonable request to the corresponding author.

## Supplementary Material

Supplemental file 1
