# Supplementary material for: Intranasal Immunization with Acellular Pertussis Vaccines Results in Long-Term Immunity to Bordetella pertussis in Mice
Source: Infect Immun. 2021 Feb 16;89(3):e00607-20. doi: 10.1128/IAI.00607-20 (PMC8097269; doi:10.1128/IAI.00607-20)
Supplement: Supplemental file 1 [file IAI.00607-20-s0001.pdf]

SUPPLEMENTAL MATERIAL

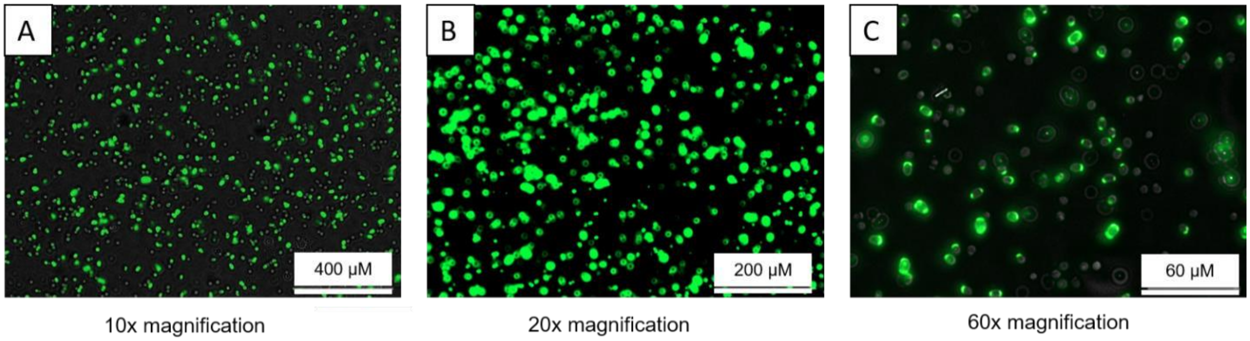

Supplementary Figure 1. Immunofluorescence microscopy of whole β-glucan particle, IRI-1501. Representative images of fluorescently labeled IRI-1501 with DTAF (5-(4,6Dichlorotriazinyl) Aminofluorescein) at (A) 10x, (B) 20x and (C) 60x magnification using a Lionheart microscope. DTAF was dissolved in cell culture grade DMSO and incubated with IRI1501 at a 1:10 ratio overnight at room temperature. IRI-1501+DTAF was washed and resuspend in PBS at a dilution of 1:100.

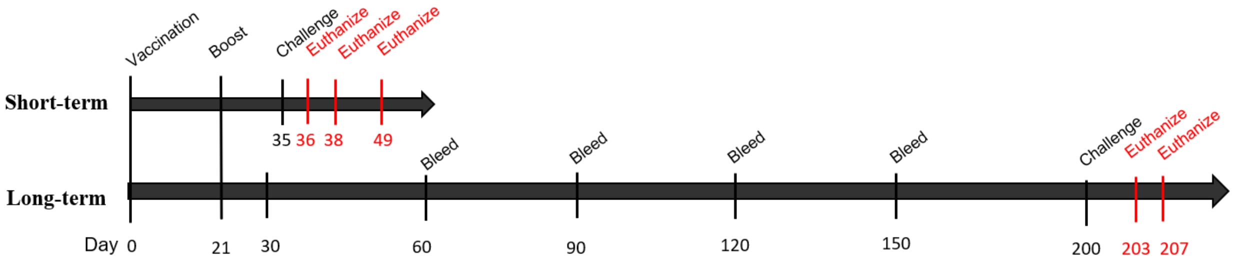

Supplementary Figure 2. Short- and long-term vaccination, boost and challenge models. BALB/c mice were used in both the short- and long-term studies and mice were vaccinated at day 0 and boosted at day 21 day in both studies. Mice were challenged at day 35 and euthanized 1, 3,

and 14 days post-challenge for the short-term study. Serum was collected monthly for the longterm study and mice were challenge 6 months post-boost and then euthanized 3- and 7-days post-challenge.

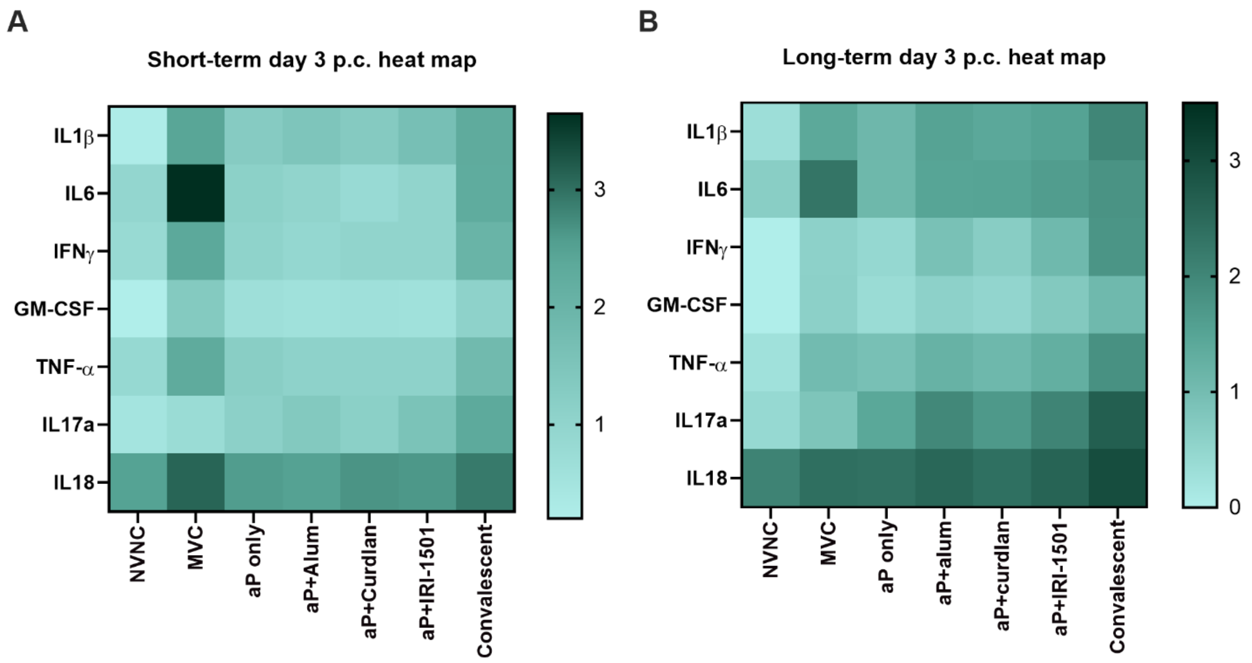

Supplementary Figure 3. Heat map of pulmonary pro-inflammatory cytokines at 3 days postchallenge. (A) Short-term and (B) long-term study cytokine response in the lung supernatant determined using a multiplex immunoassay kit. Heat map intensity scale represents log of pg/mL. NVNC=non-vaccinated non-challenge; MVC=mock-vaccinated and challenged; p.c.=post-challenge.

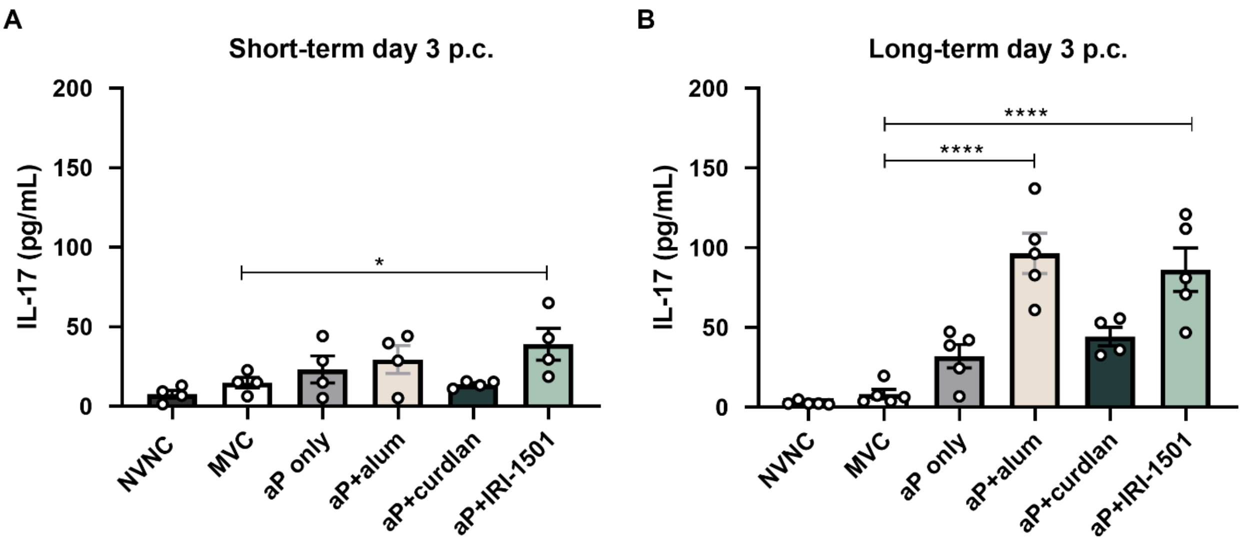

Supplementary Figure 4. Levels of pulmonary IL-17a (pg/mL) at 3 days post-challenge determined by multiplex immunoassay kit in short- (A) and long-term (B) studies. The asterisks indicate a significant difference from MVC. Data presented as means ± SEM, *p* values were determined by one-way ANOVA with Dunnett's post-hoc test compared to mock vaccinated mice for statistical analysis. *p<0.05, ****p<0.001 indicates significance from MVC. NVNC=nonvaccinated non-challenge; MVC=mock-vaccinated and challenged; p.c.=post-challenge.

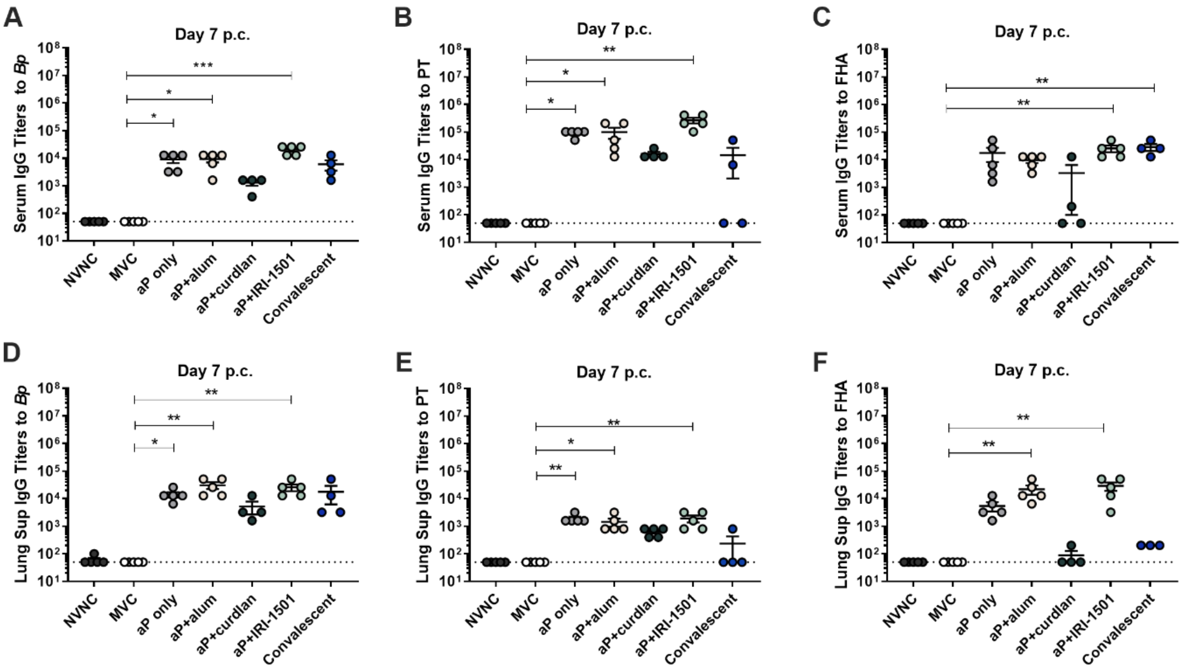

Supplementary Figure 5. Anti-*B. pertussis* (*Bp*), anti- PT and anti-FHA in the serum (A, B, C) and lung supernatant (D, E, F) at 7 days post-challenge for the long-term study were detected by ELISA assay. Data presented on a log scale as means ± SEM, n = 5 per treatment group. Kruskal Wallis test used for statistical analysis. Dotted line indicates the lowest limit of detection. *$p<0.05$, **$p<0.005$, ***$p<0.001$ indicates significance from MVC. NVNC=non-vaccinated non-challenge;

MVC=mock-vaccinated and challenged; p.c.=post-challenge.

38

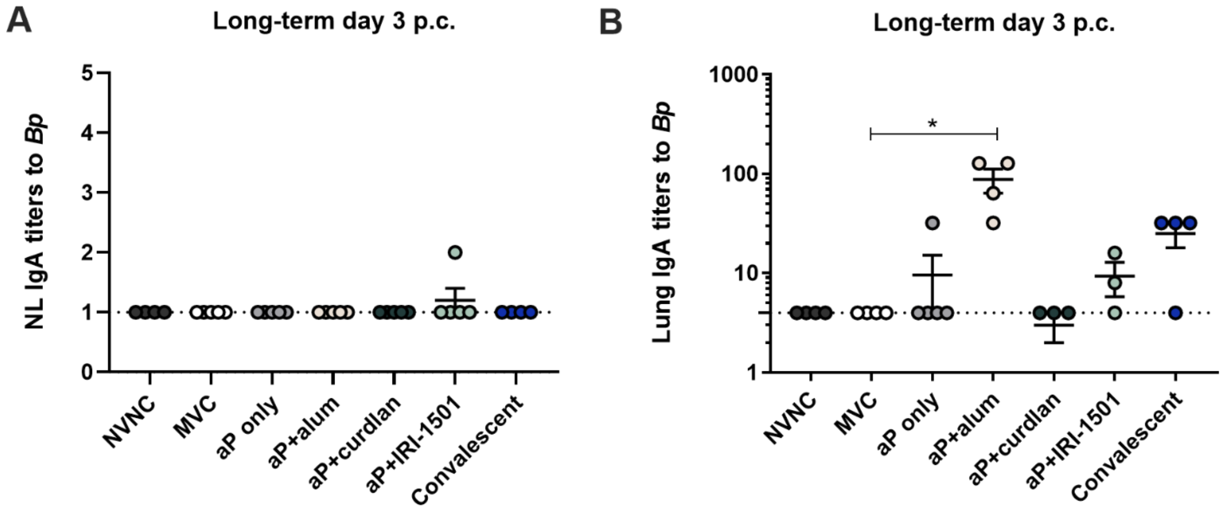

Supplementary Figure 6. In the long-term study, anti-*B. pertussis* IgA titers at 3 days postchallenge in the nasal lavage and lung determined by ELISA assay. (A) Nasal lavage anti- *B. pertussis* IgA antibody titers 3 days post-challenge. (B) Pulmonary anti-*B. pertussis* IgA titers 3 days post-challenge. Results shown as mean ± SEM ($n = 5$). *p values* were determined by KruskalWallis with Dunnett’s post-hoc test. * $p<0.05$ indicates significant difference from MVC. NL=nasal lavage; NVNC=non-vaccinated non-challenge; MVC=mock-vaccinated and challenged; p.c.=post-challenge.

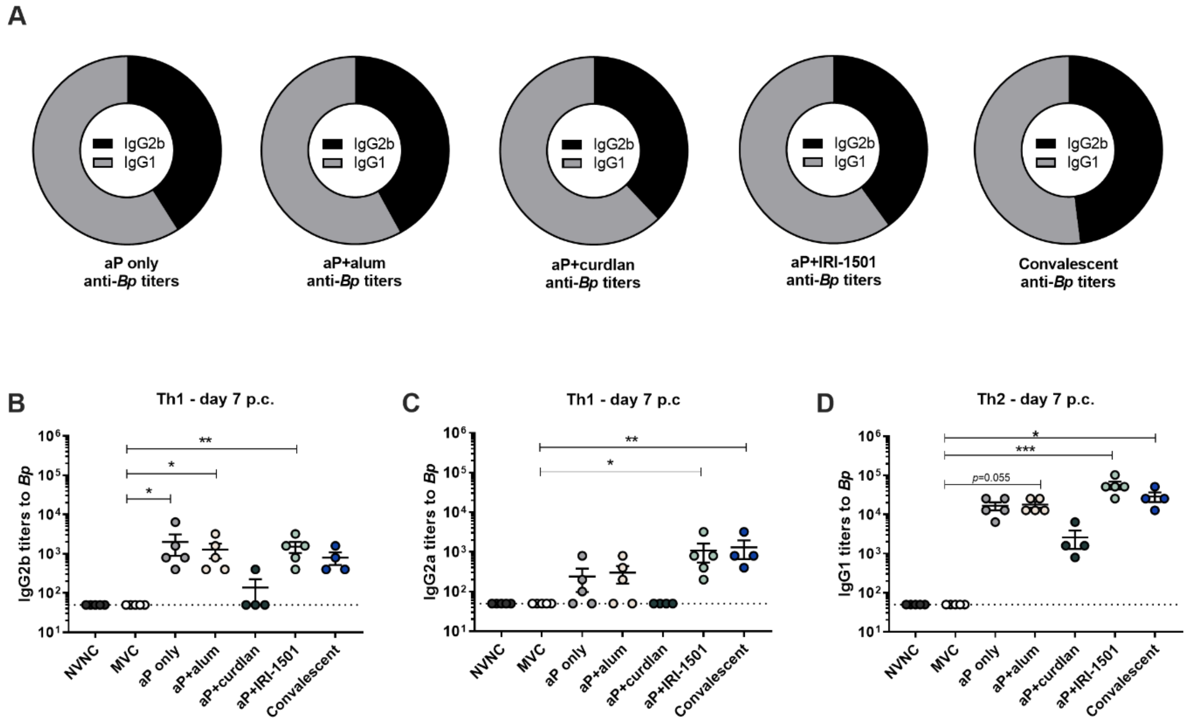

Supplementary Figure 7. In the long-term study, anti-*B. pertussis* Th1/Th2 IgG antibody response in the serum at 7-days post-challenge determined by ELISA. (A) Ratio of anti-*B. pertussis* IgG2b (Th1) to anti-*B. pertussis* IgG1 (Th2) in the serum 7 days post-challenge. Anti-*B. pertussis* (B) IgG2b, (C) IgG2a, and (D) IgG1 titers 7 days post-challenge. Results shown as mean ± SEM ($n = 5$). Dotted line indicates the lowest limit of detection. *$p<0.05$, **$p<0.005$, ***$p<0.001$ indicates significance from MVC. NVNC=non-vaccinated non-challenge; MVC=mock-vaccinated and challenged; p.c.=post-challenge.

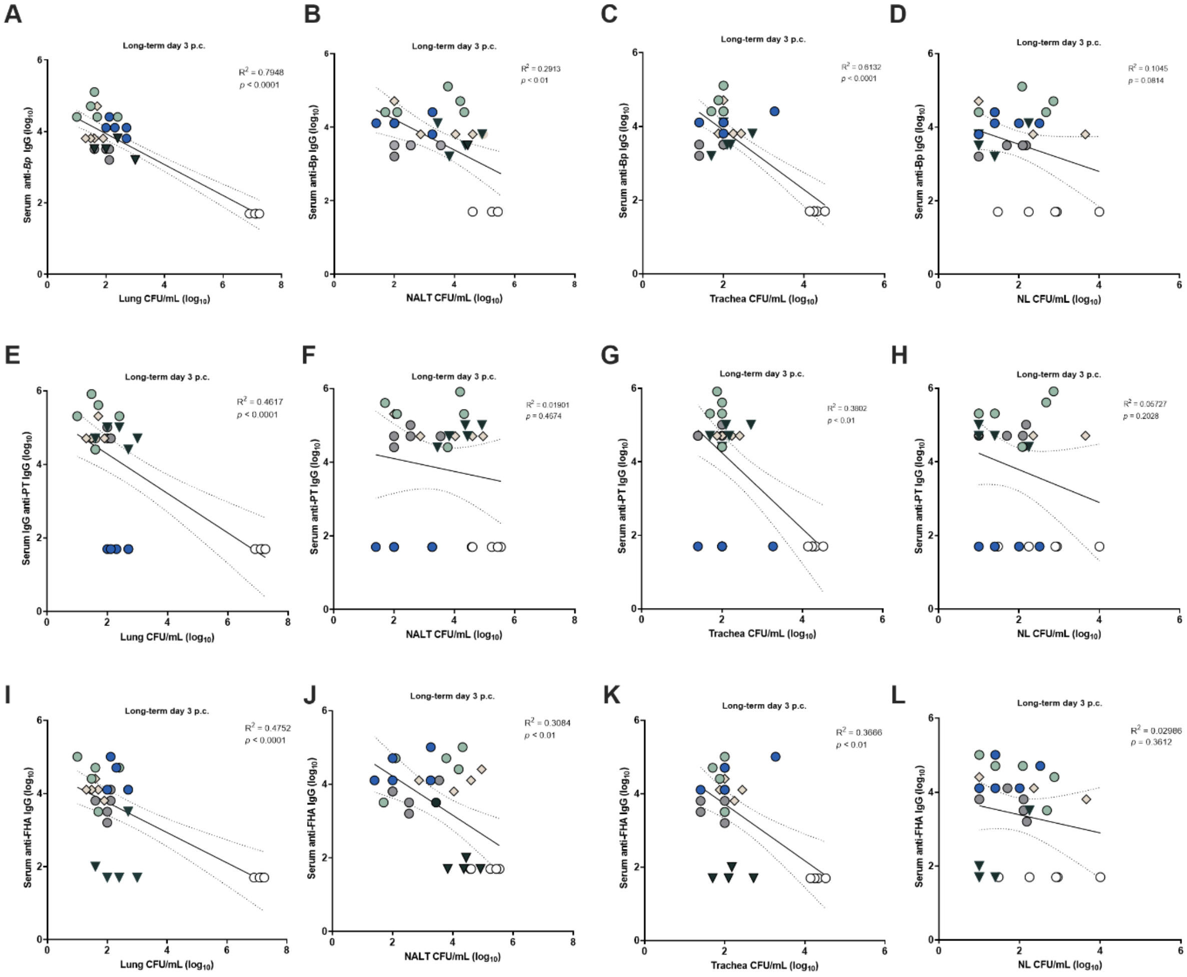

Supplementary Figure 8. Linear regression from the long-term study of *B. pertussis* (*Bp*) or antigen specific IgG titers in the serum against bacterial burden in the lung, NALT, trachea, or nasal lavage. Serum anti-*B. pertussis* IgG to (**A**) lung, (**B**) NALT, (**C**), trachea, or (**D**) nasal lavage CFUs. Serum anti-PT IgG to (**E**) lung, (**F**) NALT, (**G**) trachea, or (**H**) nasal lavage CFUs. Serum anti-FHA IgG to (**I**) lung, (**J**) NALT, (**K**) trachea, or (**L**) nasal lavage CFUs. White circle: MVC; Grey circle: aP only; Tan diamond: aP+alum; Forest green upside down triangle: aP+curdlan; Light green: circle aP+IRI-1501; Cobalt circle: convalescent group. NVNC=non-vaccinated nonchallenge; MVC=mock-vaccinated and challenged; p.c.=post-challenge.

Supplementary Table 1. Tissue-resident memory T (T_{RM}) and Tissue- effector memory (T_{EM}) panel for flow cytometry.

Antibody	Fluorophore	Company	Catalog Number
**CD3e**	BV510	BD Biosciences	563024
**CD4**	APC-Cy7	Biolegend	100526
**CD44**	BB515	BD Biosciences	564587
**CD62L**	APC	BD Biosciences	553152
**CD69**	BV421	BD Biosciences	562920
**CD103**	APC-R700	BD Biosciences	565529
